# Color and tone color: audiovisual crossmodal correspondences with musical instrument timbre

**DOI:** 10.3389/fpsyg.2024.1520131

**Published:** 2025-01-07

**Authors:** Lindsey Reymore, Delwin T. Lindsey

**Affiliations:** ^1^School of Music, Dance and Theatre, Herberger Institute for Design and the Arts, Arizona State University, Tempe, AZ, United States; ^2^School of Music, The Ohio State University, Columbus, OH, United States; ^3^Department of Psychology, The Ohio State University, Columbus, OH, United States; ^4^College of Optometry, The Ohio State University, Columbus, OH, United States

**Keywords:** timbre, color, crossmodal correspondences, timbre semantics, music and color

## Abstract

Crossmodal correspondences, or widely shared tendencies for mapping experiences across sensory domains, are revealed in common descriptors of musical timbre such as *bright*, *dark*, and *warm*. Two experiments are reported in which participants listened to recordings of musical instruments playing major scales, selected colors to match the timbres, and rated the timbres on crossmodal semantic scales. Experiment A used three different keyboard instruments, each played in three pitch registers. Stimuli in Experiment B, representing six different orchestral instruments, were similar to those in Experiment A but were controlled for pitch register. Overall, results were consistent with hypothesized concordances between ratings on crossmodal timbre descriptors and participants’ color associations. Semantic ratings predicted the lightness and saturation of colors matched to instrument timbres; effects were larger when both pitch register and instrument type varied (Experiment A) but were still evident when pitch register was held constant (Experiment B). We also observed a weak relationship between participant ratings of musical stimuli on the terms *warm* and *cool* and the warmth-coolness of selected colors in Experiment B only. Results were generally consistent with the hypothesis that instrument type and pitch register are related to color choice, though we speculate that these associations may only be relevant for certain instruments. Overall, the results have implications for our understanding the relationship between music and color, suggesting that while timbre/color matching behavior is in many ways diverse, observable trends in strategy can in part be linked to crossmodal timbre semantics.

## 1 Introduction

Musical sounds are often described using terms common in other sensory domains; for example, sounds may be bright (visual), sweet, (gustatory), or rough (tactile). Crossmodal correspondences, as distinct from synesthesia, refer to tendencies in the general population for certain stimuli or features of stimuli in one sensory domain to be associated with stimuli or features in another sensory domain (Spence and Sathian, 2020; see also Deroy and Spence, 2013; Motoki et al., 2023; Parise and Spence, 2013; Spence, 2011). These tendencies manifest in both literary and vernacular language, and previous research provides evidence consistent with the theory that crossmodal correspondences are relevant to perception as well as language (Marks, 1996; Marks, 2013). Complementary to previous literature on audio-visual crossmodal correspondences (see Spence, 2020b for a review), which has primarily focused on the basic feature of pitch (e.g., Martino and Marks, 1999; Walker et al., 2012) or on complex musical compositions as color-evocative (see Spence, 2020a), the experiments reported in this paper address correspondences between color and musical instrument timbre, engaging the question of how these correspondences may be reflected in crossmodal semantic timbre descriptors.

The documented widespread use of crossmodal terms in timbre lexicons (e.g., Saitis and Weinzierl, 2019; Wallmark, 2019a; Reymore and Huron, 2020) suggests that timbre-color correspondences are a promising area of investigation. Saitis et al. (2020) argue that studying crossmodal correspondences with timbre offers a new way of approaching questions about the mechanisms of auditory semantics, while at the same time, providing more general insight into timbre perception and human semantic processing. Relatively few previous studies have considered timbre as a contributing feature to crossmodal correspondences between sound/music and color. Adeli et al. (2014) asked participants to match timbres to colored shapes and found that the “softer” timbres of marimba and piano were associated with blue and green shapes, while the “harsher” timbres of crash cymbals, gong, and triangle were matched with red or yellow shapes. An experiment by Reuter et al. (2018) tested associations between single note samples at varying pitch heights from a variety of Western musical instruments and found that selected colors appeared to be related to both pitch height and spectral features, while also identifying a few associations between hue and instrument. Participants in Qi et al. (2020) selected sounds produced by various Chinese instruments to match given colors; results suggested significant pitch-color associations as well as some select instrument timbre-color associations. Though these studies address color and timbre, they do not investigate the relevance of crossmodal linguistics. A recent study by Gurman et al. (2021), which explores correspondences between timbre and shape, demonstrates the potential for semantic tasks to supplement assessments of crossmodal correspondences. The authors argue that parallel semantic tasks can augment our understanding of crossmodal correspondences, offering insight into participants’ interpretation of the meaning of stimuli.

The present experiments were designed to examine the relationships between timbre-color associations and crossmodal semantic ratings. Participants first listened to a set of recordings of musical instruments presented in random order. Following each recording, they selected from a color palette all those colors that they felt were most consistent with the sound quality or characteristic of the musical instrument. Participants then listened to the recordings again in a random order, this time rating the degree to which each musical stimulus was associated with various crossmodal timbre descriptors. The principal aim of this study is to map relationships between people’s use of crossmodal timbre descriptors and their individual color choices, assessing how closely participants’ conceptions of timbral characteristics align with their corresponding color choices. This approach can be distinguished from prior research, which has analyzed timbre-color correspondences using computationally derived audio descriptors (Lindborg and Friberg, 2015; Reuter et al., 2018) or researcher-generated descriptions (Adeli et al., 2014).

Close interactions between timbre and pitch height merit careful consideration in timbre research. Although timbre contributes to sound source identification, a single sound source can produce many timbres (Siedenburg and McAdams, 2017), and timbre varies notably across the pitch range of musical instruments (Reymore, 2021; Reymore et al., 2023). Furthermore, changes in fundamental frequency (F0) have been shown to affect perceived timbral brightness (Marozeau and de Cheveigné, 2007). Many previous crossmodal experiments have used carefully controlled stimuli to attempt to isolate fundamental frequency and its perceptual correlate, pitch height, as parameters. However, pitch height and timbre are usually inextricable in everyday musical contexts: as pitch height changes, so does timbre. Our experimental design frames instrument type and pitch height as two sources of timbral variation, recognizing that pitch height and timbre cannot be fully disentangled in ecologically valid contexts. In Experiment A, we systematically vary both pitch height and instrument type, whereas Experiment B holds pitch height constant and offers comparison across a more timbrally diverse set of instruments.

To formulate hypotheses about lightness and saturation of color choices, we began by assembling an initial set of crossmodal terms that (1) have been previously identified in scholarship as common descriptors of timbre and (2) have been implicated in prior studies on audio-visual crossmodal research. We then expanded the initial set of descriptors by extrapolating based on prothetic (magnitude-related) relationships and polar alignments among the terms (see Spence, 2011). The complete set of 12 timbre descriptors rated in the experiments includes the terms *high*, *low*, *bright*, *dark*, *small*, *big*, *light in weight*, *heavy*, *happy*, *sad*, *warm*, and *cool*.

We hypothesized the first 10 terms in the above list to predict the lightness and saturation of matched colors, whereas we hypothesized *warm* and *cool* to predict the warmth-coolness of matched colors. In the following sections, we review literature motivating our selection of semantic terms.

### 1.1 Brightness, lightness, and pitch

The terms *bright* and *dark*, used commonly to describe colors, are also widely acknowledged as some of the most frequently used timbre descriptors, and the relationship between visual and timbral brightness has been investigated through numerous perceptual studies. For example, Wallmark et al. (2021) observed via a speeded classification task that incongruity between visual and timbral brightness increased error rate, though this did not affect response time; Saitis and Wallmark (2024) observed that timbral brightness modulated the perception of pitch and possibly visual brightness. More generally, past research has provided robust evidence for both pitch-brightness and timbre-brightness correspondences (e.g., Marks, 1982; Marks et al., 1987; Wallmark, 2019b; Wallmark and Allen, 2020). While the mechanisms behind such congruences are not fully understood, the audio-visual connection is readily apparent.

A relationship between pitch height and lightness is also well-documented: higher pitches are associated with lighter colors, while lower pitches are associated with darker colors (Marks, 1974; Marks, 1987; Marks, 1989; Melara and Marks, 1990; Martino and Marks, 1999; Mondloch and Maurer, 2004; Ward et al., 2006). Given that timbral variation can affect perception of pitch height (e.g., Warrier and Zatorre, 2002; Kuang et al., 2016), we included the terms *high* and *low* among our timbre semantic descriptors. We anticipated potential differences in semantic judgments on these terms even as pitch register is held constant (as in Experiment B)—and that these differences would influence lightness of color choice. Taken together, previous research on relationships among pitch, timbre, visual lightness, and visual brightness indicates that timbres perceived as *brighter* and *higher* will tend to be matched with lighter colors, whereas *darker-and lower*-sounding timbres will tend to be matched with darker colors.

### 1.2 Sound-size symbolism

The terms *big*, *small*, *heavy*, and *light (in weight)* are colloquially used in describing instrument timbre (Reymore and Huron, 2020). Previous research has not directly explored correspondences between timbral characteristics and size or weight; however, we can extrapolate predictions from past experiments that have demonstrated robust pitch-size associations, where lower sounds are linked to larger size (Walker and Smith, 1984; Mondloch and Maurer, 2004; Gallace and Spence, 2006; Evans and Treisman, 2010; Bien et al., 2012; Walker et al., 2012).

Given (1) the close connections described in the previous section among visual lightness/brightness, timbral brightness, and pitch height, and (2) the observation that contrasts of *bright-dark, high-low, light–dark,* and *small-big* are considered to be examples of polar dimensions that align with one another (Parise and Spence, 2013), we included the terms *small*, *big*, *heavy*, and *light in weight* among our semantic descriptors. Triangulating timbre-brightness, pitch-brightness, and pitch-size correspondences led us to hypothesize that timbres judged to be *smaller* and *lighter in weight* will be matched to lighter colors, whereas *bigger* and *heavier* timbres will be matched to darker colors.

Across work on audiovisual crossmodal correspondences, the role of saturation has been studied less frequently than lightness. Here, research on heaviness, saturation, and pitch guided our predictions. Alexander and Shansky (1976) observed that participants assigned heavier weights to darker and more saturated colors, while Walker et al. (2017) found that objects perceived as heavier were judged to be darker and to make lower-pitched sounds. From here, we used prothetic relationships and polar alignments to extrapolate to other terms in our set. For example, Walker and Smith (1984) found *happy* to align with *high*, *little*, and *light (in weight)* in an interference task; similarly, Eitan and Timmers (2010) observed that *light in weight-heavy* were consistently matched with *high-low* for both English-and Hebrew-speaking participants. Based on these findings, we hypothesized that timbres rated as *heavier* would not only be matched to darker colors, but to more saturated colors, guided principally by the findings of Alexander and Shansky (1976). In parallel, we anticipated *big* would also correspond to more saturated colors, whereas *small* and *light (in weight)* would map to less saturated colors.

Notably, an argument could also be made in favor of a relationship with saturation in the opposite direction, based on other previous studies. From a series of experiments using the implicit associations test, Anikin and Johansson (2019) found saturation to be positively associated with frequency and spectral centroid. Hamilton-Fletcher et al. (2017) asked participants to adjust hue and chroma to heard sounds while luminance was held constant. They similarly found that frequency, as well as a timbral manipulation caused by adjusting the spectral center of gravity, was positively correlated with saturation.

### 1.3 Emotion

Open-ended interviews suggest that musicians readily use emotion-related words in timbral discourse (Reymore and Huron, 2020). Di Stefano (2023) presents an account of how timbre can be associated with emotional qualities via the concept of atmosphere, which has an essential affective component. He notes that the relationship between timbre and perceived emotion does not depend on high-level cognitive mediation but rather is motivated by acoustic features. Indeed, behavioral evidence implicates instrument timbre in ratings of valence, tension arousal, and energy arousal, with increases on these scales corresponding to changes in acoustical descriptors such as fundamental frequency and spectral centroid (e.g., McAdams et al., 2017; Eerola et al., 2012; Korsmit et al., 2023; see also Korsmit et al., 2024).

Spence and Di Stefano (2022) argue for an emotional mediation account of color-sound correspondences, positing that ultimately, emotion explains these correspondences, “no matter whether the stimuli are simple or complex” (p. 30). Palmer et al. (2013) found strong evidence consistent with an emotion mediation account in the context of matching excerpts of classical orchestral music to color. The emotion mediation hypothesis has been supported by findings from subsequent studies using single-line piano melodies (Palmer et al., 2016), excerpts from J.S. Bach’s *Well-Tempered Clavier* (Isbilen and Krumhansl, 2016), and musical excerpts from a variety of genres (Whiteford et al., 2018). Should emotion mapping be the primary mechanism at work in timbre-color matching when musical content is controlled, we expect to observe ratings on the emotion words *happy* and *sad*, as semantic descriptions of timbre, to explain as much as or more variance in color choice as the other words in our set.

### 1.4 Warm and cool

From previous research in timbre semantics, two other descriptors stand out as ostensibly related to color associations: *warm* and *cool*. With respect to timbre description, *warm* is one of the most common descriptors in English and has been included in dimension labels across many timbre studies (see Saitis and Weinzierl for a review). *Cool*, or *cold*, as timbre descriptors, are far less common, but these terms do also emerge in timbre description (Reymore and Huron, 2020). In everyday parlance, these words are most commonly used to describe temperature, but *warm* and *cool* are also applied readily to colors, with *warm* suggesting colors like red, orange, and yellow, and *cool* suggesting colors like green and blue (likely related to temperature-associated imagery like fire and ice). This shared vocabulary across visual and auditory modalities suggests a potential association between warm and cool sounds and warm and cool colors; however, no previous studies have investigated this correspondence.

### 1.5 Summary

In sum, to generate hypotheses about relationships between timbre description and timbre-color matching, we drew on common crossmodal timbre terms, choosing specific terms related to previously established correspondences between pitch-brightness/lightness, timbre-brightness, pitch-size/weight, and music-emotion. Motivated by the literature reviewed above, we assembled a set of ten terms that were used to predict both lightness and saturation of colors matched to timbres. Our experiments also address whether the terms *warm* and *cool*, often used to describe both timbre and color, are related in a crossmodal context.

## 2 Hypotheses

We report the results of two experiments in which participants rated instrument timbres on 12 crossmodal semantic descriptors and then matched the same heard excerpts with colors. Specifically, these experiments tested hypothesized relationships between individual semantic terms and the lightness, saturation, and warmth-coolness of the matched color choices. Experiment A systematically varied pitch register across three different keyboard instruments; Experiment B tested a more diverse set of six different musical instruments while holding pitch register constant.

The principal aim of this research is to examine relationships between crossmodal semantic descriptions of timbre and timbre-color associations. The experimental design also presented the opportunities to assess the relevance of sound source identity (musical instrument) and fundamental frequency to timbre-color associations and to explore trends in timbre-color matching across a range of musical instruments.

*H1*: When asked to match timbres with colors, participants’ semantic assessments of timbres will be systematically associated with color choices.

*H1a*: Semantic ratings on the terms *bright*, *dark*, *high, low*, *light in weight*, *heavy*, *small*, *big*, *happy*, and *sad* will predict the lightness of matched colors. Specifically, ratings on the terms *bright, high, light in weight, small,* and *happy* will be positively associated with lightness; ratings on *dark*, *low*, *heavy*, *big*, and *sad* will negatively correlate with lightness.

*H1b*: Semantic ratings on the terms *bright*, *dark*, *high, low*, *light in weight*, *heavy*, *small*, *big*, *happy*, and *sad* are associated with saturation of matched colors. Specifically, ratings on the terms *bright, high, light in weight, small,* and *happy* will be negatively associated with saturation; ratings on *dark*, *low*, *heavy*, *big*, and *sad* will positively correlate with saturation.

*H1c*: Semantic ratings on the terms *warm* and *cool* are associated with perceived warmth-coolness of matched colors.

*H2*: When asked to match timbres with colors, lightness, saturation, and warmth-coolness of participants’ color choices will vary systematically across different musical instrument timbres.

*H3*: When asked to match timbres with colors, lightness, saturation, and warmth-coolness of participants’ color choices will vary systematically with pitch register. Higher pitch register will result in increased lightness and decreased saturation.

## 3 Experiment A

### 3.1 Methods

#### 3.1.1 Participants

Participants (*n* = 96; 41 M, 52 F, 1 agender, 2 not reporting) were recruited from the Center for Science and Industry (COSI) in Columbus, Ohio, USA. Participant age ranged from 18 to 70 (*M* = 30.2; *SD* = 11.0). Three participants reported synesthesia, including one color-sound, one color-key, and one not specified; nine participants indicated they were not sure whether they experienced synesthesia. We did not exclude data from these participants.

#### 3.1.2 Stimuli

##### 3.1.2.1 Recordings

Recordings were made on three different keyboard instruments using a Zoom H4N recorder: a Blüthner grand piano, built in 1906, a Flemish harpsichord after the Colmar Ruckers 1624 original, built by Keith Hill, Op. 486, 2016, and a Lautenwerk or lute harpsichord, built by Keith Hill, Op. 510, 2018. The lute harpsichord uses gut strings, while the Flemish harpsichord uses metal; this difference results in the former instrument producing a softer, more mellow sound and the latter producing a brighter, more nasal sound. Recordings were made on-site at Hill’s workshop. The recording level was kept constant for all recordings, and the microphone was placed at roughly equal distances away from the instruments. All instruments were tuned within the half hour prior to their recordings. Three ascending major scales were recorded on each of the three instruments, beginning on F2, F3, and F4. Performers referred to a silent, blinking metronome, set at 88 beats per minute, to maintain a consistent tempo, holding each pitch for two beats. By using complete major scales as stimuli in the experiments reported here (as opposed to single notes or composed music) we consider stimuli that while for the most part simple, cover part of the middle ground between simple and complex, conferring an additional dimension of musicality while maintaining control of the musical content, which in composed music may introduce numerous confounding variables.

##### 3.1.2.2 Color palette

**Figure 1:**
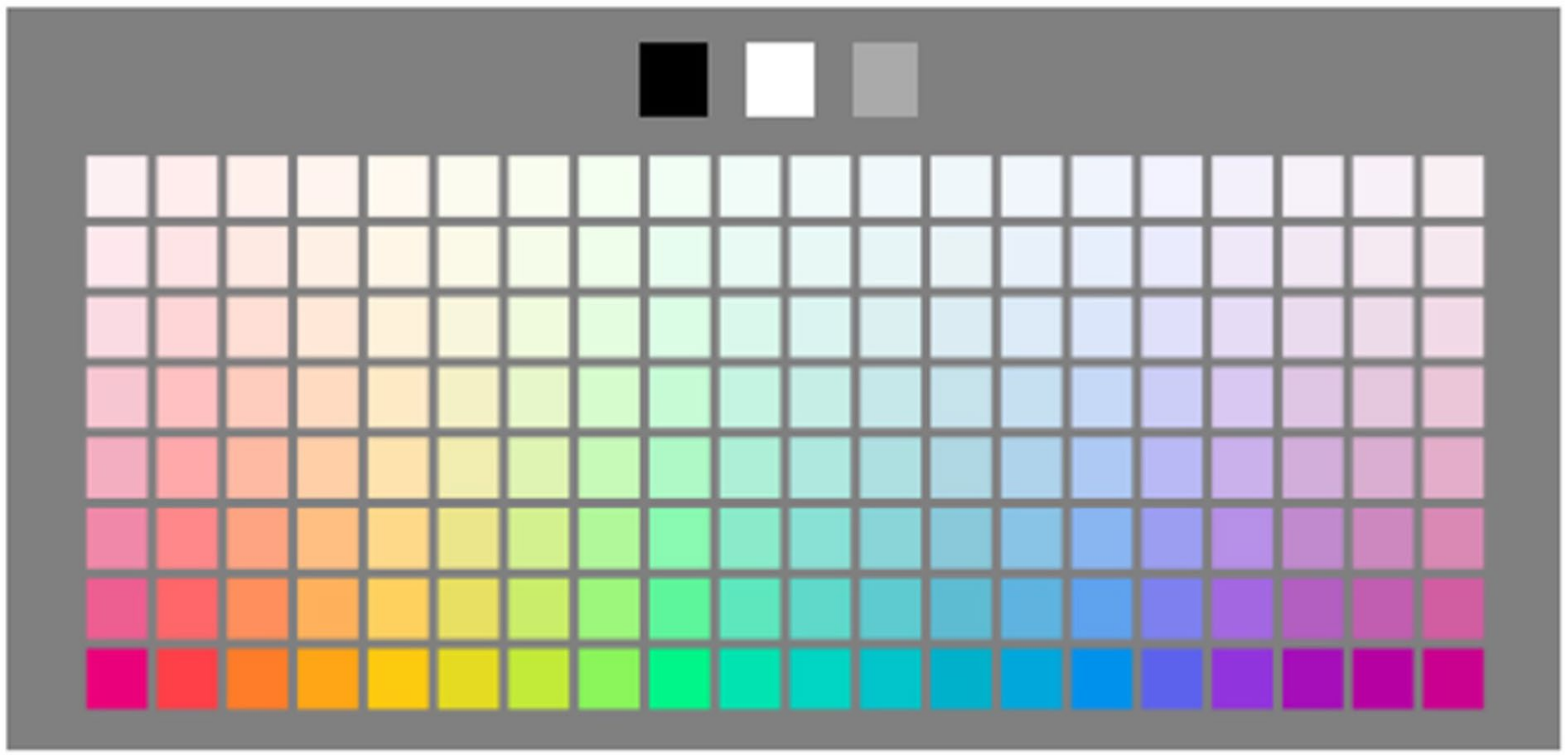
Color palette used in Experiments A and B. Colors are arranged in 20 × 8 matrix of chromatic colors plus black, white and gray. Here, hue varies horizontally left-to-right from reds through greens and blues to violets; hue in any column is constant but saturation increases from top to bottom. In order to minimize bias in color selections due to unintended configural cues in the palette, hues were rotated randomly from trial-to-trial and rows were “flipped” so that on some trials, saturation increased bottom to top.

An approximation of the color palette used for our control study and all subsequent experiments described below is shown in Figure 1. The colors, which averaged 258 cd/m^2^ in maximum luminance, were spatially arranged in a 20 × 8 palette of color samples organized according to hue (palette columns spanning the color circle) and saturation from most saturated to almost white, displayed on an 85 cd/m^2^ 5000K gray background. Also included in each display were black, white, and gray samples located in a row immediately above the color palette.

Colors for elements in the bottom row of the color palette as oriented in Figure 1 were chosen to correspond to high-chroma hues spanning the color circle and to include examples of the lexical categories red, green, blue, yellow, orange and purple. Greens and blue-greens, and blues were somewhat less saturated than other colors, while blues were also somewhat less luminous than the other colors. Variations in saturation and luminance were dictated by constraints on color reproduction imposed by the iPad display’s color gamut (P3). Finally, yellows were somewhat more luminous than average, as less luminous yellows were informally judged by us to be poor examples of this color category.

Once the bottom row of color palette colors had been established, the colorimetric purities of colors in subsequent rows were chosen to produce approximately equal steps in saturation for each hue from its maximum (bottom row) to a minimal (top row) difference from white (see Wyszecki and Stiles, 1982 for formal definitions). The colorimetric purities of the minimum-saturation colored samples were chosen to create predominantly white samples, slightly tinged with the corresponding hue for that column.

Participants had access to a slider simulated on the iPad touch screen that they could use to manually adjust the mean luminance of the color palette in a quasi-continuous fashion before making their color sample selections. As the slider was moved, the resulting color samples maintained their respective chromaticities; only the luminances of the samples relative to their respective maxima changed.

To address the concern that absolute spatial correspondences in the arrangement of palette colors could influence color choice (for example, less saturated samples always appearing toward the top of the palette), we introduced a random trial-to-trial variation in the absolute spatial arrangement of the palette samples displayed on the iPad. For example, on some trials, sample saturation increased from top-to-bottom, as shown in Figure 1, while on other trials, saturation increased from bottom-to-top. Additionally, mapping of the hue circle onto the matrix shown in Figure 1 was randomly rotated from one trial to the next, while preserving hue order.

#### 3.1.3 Procedure

Following a brief demographic survey, participants used a custom-programmed and calibrated iPad and headphones to listen to the musical stimuli and view the visual stimuli. In the first part of the experiment, participants were asked to listen to each excerpt and then to choose the color or colors that they felt best represented the sound quality and character of the musical instrument. Participants were provided with an array of colors (Figure 1). They were instructed to set the slider to the desired lightness and then use the touch screen of the iPad to select a single color or multiple colors, with no limit on the number of colors that could be selected.

In the second part of the experiment, participants listened to the same excerpts in a different random order and were asked to rate the appropriateness of various adjectives for describing the quality and character of the sound, including *high, low, bright, dark, light in weight*, *heavy*, *small, big, happy, sad, warm* and *cool,* using a continuous slider that ranged from “not appropriate” to “very appropriate.” The presentation order of the adjectives and of stimuli were randomized.

### 3.2 Results

**Figure 2:**
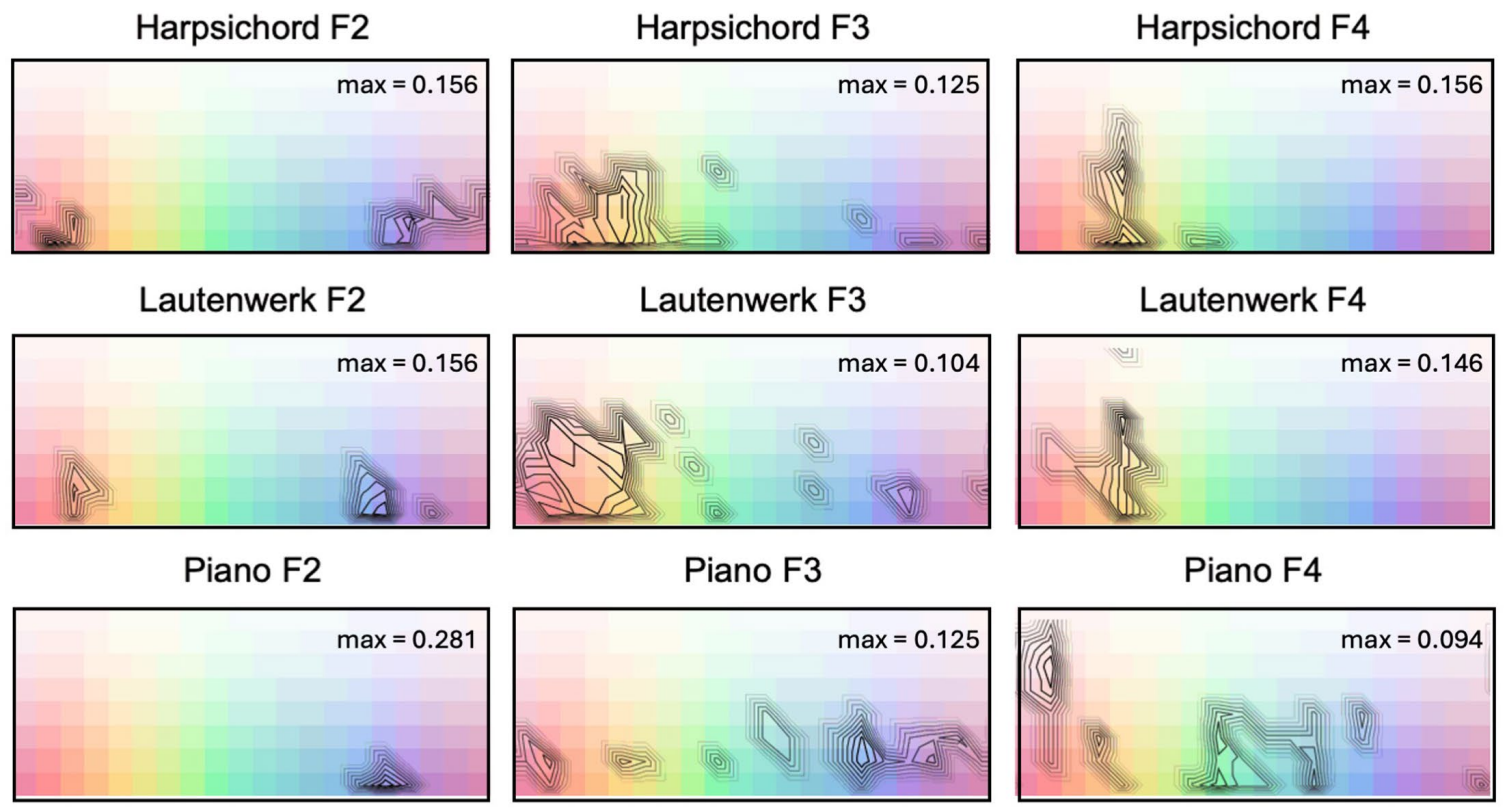
Contour plots of relative consensus in color selections (irrespective of luminance settings) for keyboard instruments played in each of three pitch registers (F2, F3, F4) in Experiment A. Maximum consensus for each instrument/pitch condition is indicated in the upper-right corner of each panel. Contours have been adjusted in contrast to highlight regions of color plots with highest consensus values.

Consensus plots (Figure 2) demonstrate relative agreement across subjects in their color selections for each keyboard instrument and pitch register tested in Experiment A; these plots map onto the palette used in Experiment A, as shown in the figure. Each palette shows contour plots of consensus relative to the maximum consensus for that instrument. The maximum consensus, indicated in the upper-right corner of each panel, is expressed as the proportion of participants selecting the most frequently-chosen color square. Note that participants were able to select multiple color squares. In Experiment A, the median number of color samples chosen for a given stimulus was 4 (IQR = 6), with a minimum of 1 and a maximum of 44.

Stimuli in the lowest octave (F2) were concentrated toward greater saturation levels with selections moving toward less saturation as pitch register increases, at least from F2 to F3. Overall, there was a bias toward more saturated colors. All three keyboard instruments in their lowest octaves tended to map onto blues and purples, while the Lautenwerk and the Flemish harpsichord also include a second concentration of responses in highly saturated reds, oranges, and browns. In the upper two octaves, selections for the piano show a diverse range of hues, while selections for the Lautenwerk and Flemish harpsichord show a predilection for reds, oranges, yellows, and some greens. The highest octaves in both harpsichords show higher consensus, particularly on yellows. The piano does not appear to have a unique hue profile that remains consistent as register changes; both harpsichords demonstrate concentrations of warmer responses in all three octaves, though at F2, they also demonstrate a second concentration in blues and purples that is consistent across the three instruments.

### 3.3 Analysis

To assess relationships between semantic descriptors and color choices (H1), we constructed three sets of regression models to predict color-related dependent variables (lightness, saturation, warmth-coolness) from semantic ratings of individual terms. The effects of musical instrument (H2) and pitch register (H3) on color choices were tested via three two-way ANOVAs with lightness, saturation, and warmth-coolness as dependent variables.

#### 3.3.1 Semantic ratings (H1)

To test hypothesized relationships between specific semantic terms and the lightness, saturation, and warmth-coolness of color choices, we evaluated significance for each semantic term with separate models. Modeling individual terms allowed us to (1) identify which terms have significant relationships to particular dimensions of matched colors and (2) quantify relative effect sizes.

In sum, three sets of models were built to test H1. The purpose of the first set was to assess the relationship of semantic terms with lightness (H1a); this set included ten models, one for each hypothesized term. Here, lightness was predicted from semantic ratings on the given word, as a fixed effect, with participant ID included as a random intercept. A similar process yielded the second set of models, now with saturation as the dependent variable (H1b). To test relationships between ratings on the terms *warm* and *cool* and the warmth-coolness of selected colors (H1c), we first calculated a measure of warmth-coolness (the warm-cool index), based on a control study (see Supplementary material S1). The third set of models included two models, one for *warm* and the other for *cool*, which predicted the warm-cool index from ratings on each term.

All models were built with R Statistical Software (v4.3.0, R Core Team, 2023). We noted deviations from the assumptions of normality for linear regression, the extent of which varied across the models reported in this paper. Some of these deviations appear to be related to a ceiling effect for the lightness slider. In order to be able to compare results within and across experiments, it was necessary to use the same modeling techniques throughout. As linear mixed models are easier to interpret and thus preferable to report, we opted to first model all hypothesized relationships using both parametric (linear mixed models) and nonparametric methods (cumulative link mixed models) with the intention of comparing results: should the two methods yield converging results, we would report the more easily interpretable linear mixed models. Because results from both parametric and nonparametric versions of the models were indeed highly similar, we report results of the linear mixed models in the manuscript of the paper, noting two minor discrepancies accordingly. For all models, we include plots of the distributions of the residuals, qq plots, and full parametric and nonparametric model summary details in the Supplementary materials S2–S4.

Linear mixed models were built using the lme4 package (Bates et al., 2015); cumulative link mixed models were built with the *clmm* function in the ordinal package (Christensen, 2023). Pseudo-*R^2^* values were calculated using the *r2_nakagawa* function from the performance package (Lüdecke et al., 2021). All variables were scaled and centered prior to modeling.

Finally, note that we report unadjusted *p-*values throughout the paper. Given the number of models reported in this paper, we anticipate that some results are spurious; effects with *p-*values close to 0.05 should be interpreted with caution. We make note of this where relevant in the Discussion.

##### 3.3.1.1 Lightness (H1a)

Participants were able to choose as many or as few samples as they liked; for modeling, we computed the average lightness of a participant’s chosen samples for each stimulus. A subset of participants (*n* = 13) did not use the slider when choosing colors, defaulting to the lightest setting, while others (*n* = 11) adjusted the lightness slider for only one stimulus. Because there was little or no variance on luminance for these participants, models of lightness are reported for the subset of participants (n = 72) who made use of the slider for two or more trials.

H1 predicts significant relationships between lightness values of matched colors and timbre semantic ratings on 10 terms. Correlations between participants’ ratings and corresponding lightness values were in the predicted directions: that is, higher ratings on *bright*, *high*, *light in weight*, *small*, and *happy* corresponded with lighter colors, whereas higher ratings on *dark*, *low*, *heavy*, *big*, and *sad* corresponded with darker colors.

All 10 linear mixed models, which included random intercepts for participant ID, yielded significant results for the fixed effect of semantic rating. To provide a metric of effect size that can be used to compare results within and across experiments in this paper, we calculated pseudo-*R^2^* values. Table 1 reports *p* values for the fixed effect in each model (i.e., ratings of the semantic term), along with marginal and conditional *R^2^* values for each model. Here, marginal *R^2^* represents an approximation of variance explained by fixed effects (in this case, semantic ratings for a model’s given term), whereas conditional *R^2^* represents an approximation of the total variance explained by both the fixed and random effects. These pseudo-*R^2^* values are not equivalent to *R^2^* in a standard linear model, but rather provide useful context for comparing across models within the paper and interpreting our overall results.

**Table 1:** Results of linear mixed models predicting the lightness of chosen colors from semantic ratings of 10 terms in both Experiment A and Experiment B.

Lightness ~ Semantic Rating + (1 | Participant)						
	Experiment A (Keyboards)			Experiment B (Orchestral Instruments)		
	*p*	Marginal *R^2^*	Conditional *R^2^*	*p*	Marginal *R^2^*	Conditional *R^2^*
Bright	<0.001	0.14	0.21	0.003	0.02	0.16
Dark	<0.001	0.14	0.19	0.01	0.02	0.15
High	<0.001	0.15	0.19	0.01	0.02	0.16
Low	<0.001	0.16	0.22	0.001	0.03	0.16
Light in weight	<0.001	0.15	0.23	0.04	0.01	0.16
Heavy	<0.001	0.15	0.21	<0.001	0.03	0.17
Small	<0.001	0.10	0.16	0.62	0.00	0.15
Big	<0.001	0.12	0.17	0.73	0.00	0.15
Happy	<0.001	0.09	0.14	0.02	0.01	0.16
Sad	<0.001	0.08	0.12	0.001	0.03	0.18

For each model, the table includes *p-*values of the fixed effect along with corresponding marginal and conditional *R^2^* values. For individual model summaries and additional detail, see Supplementary materials S4.3, S4.4. Summary of models predicting lightness from semantic ratings.

##### 3.3.1.2 Saturation (H1b)

In cases where participants made multiple color selections, saturation values were averaged for modeling, in parallel to our treatment of the lightness data. In deciding to use the mean for analysis, we first evaluated the data and found that color selections were almost always made in single clusters of contiguous color samples, for which the mean was a reasonable representation.

All correlations between semantic ratings and saturation were in the predicted directions, where higher ratings on *bright*, *high*, *light in weight*, *small*, and *happy* corresponded with less saturated colors, whereas higher ratings on *dark*, *low*, *heavy*, *big*, and *sad* corresponded with more saturated colors.

Structures for the linear mixed models predicting saturation were identical to those built for lightness, except that the average saturation of chosen colors replaced lightness as the dependent variable. We included all participant observations in these models, as use of the slider affected variance in lightness only.

Most models yielded a significant effect of semantic rating, with generally higher *p* values and lower pseudo-*R^2^* values in comparison to the lightness models. Ratings on the term *sad* were not significant; models for two other terms—*dark* and *small*—achieved significance when modeled using nonparametric methods, but not in linear mixed models (see Supplementary material S4.5). Generally, while most terms are significant in predicting saturation, effect sizes are small. Table 2 below reports *p* values for fixed effects, along with the marginal and conditional *R^2^* values for each model.

**Table 2:** Results of linear mixed models predicting the saturation of chosen colors from semantic ratings of 10 terms, including the *p* values of the fixed effect along with marginal and conditional *R^2^* values.

Saturation ~ Semantic Rating + (1 | Participant)						
	Experiment A (Keyboards)			Experiment B (Orchestral Instruments)		
	*p*	Marginal *R^2^*	Conditional *R^2^*	*p*	Marginal *R^2^*	Conditional *R^2^*
Bright	0.01	0.01	0.14	0.17	0.00	0.32
Dark*	0.11	0.00	0.14	0.94	0.00	0.32
High	0.01	0.01	0.14	0.15	0.00	0.32
Low	0.006	0.01	0.14	0.21	0.00	0.32
Light in weight	0.003	0.01	0.14	0.02	0.01	0.33
Heavy	0.01	0.01	0.14	0.08	0.01	0.32
Small*	0.05	0.00	0.14	0.12	0.00	0.33
Big	0.01	0.01	0.14	<0.001	0.02	0.23
Happy	0.01	0.01	0.14	0.06	0.01	0.32
Sad	0.25	0.00	0.14	0.27	0.00	0.33

For individual model summaries and additional detail, see Supplementary materials S4.5, S4.6. Summary of models predicting saturation from semantic ratings. *Among the saturation models, the terms *dark* and *small* did not reach significance in the linear mixed models but are significant when modeled using cumulative link mixed models. See Supplementary material S4.5.

##### 3.3.1.3 Warm-cool (H1c)

**Figure 3:**
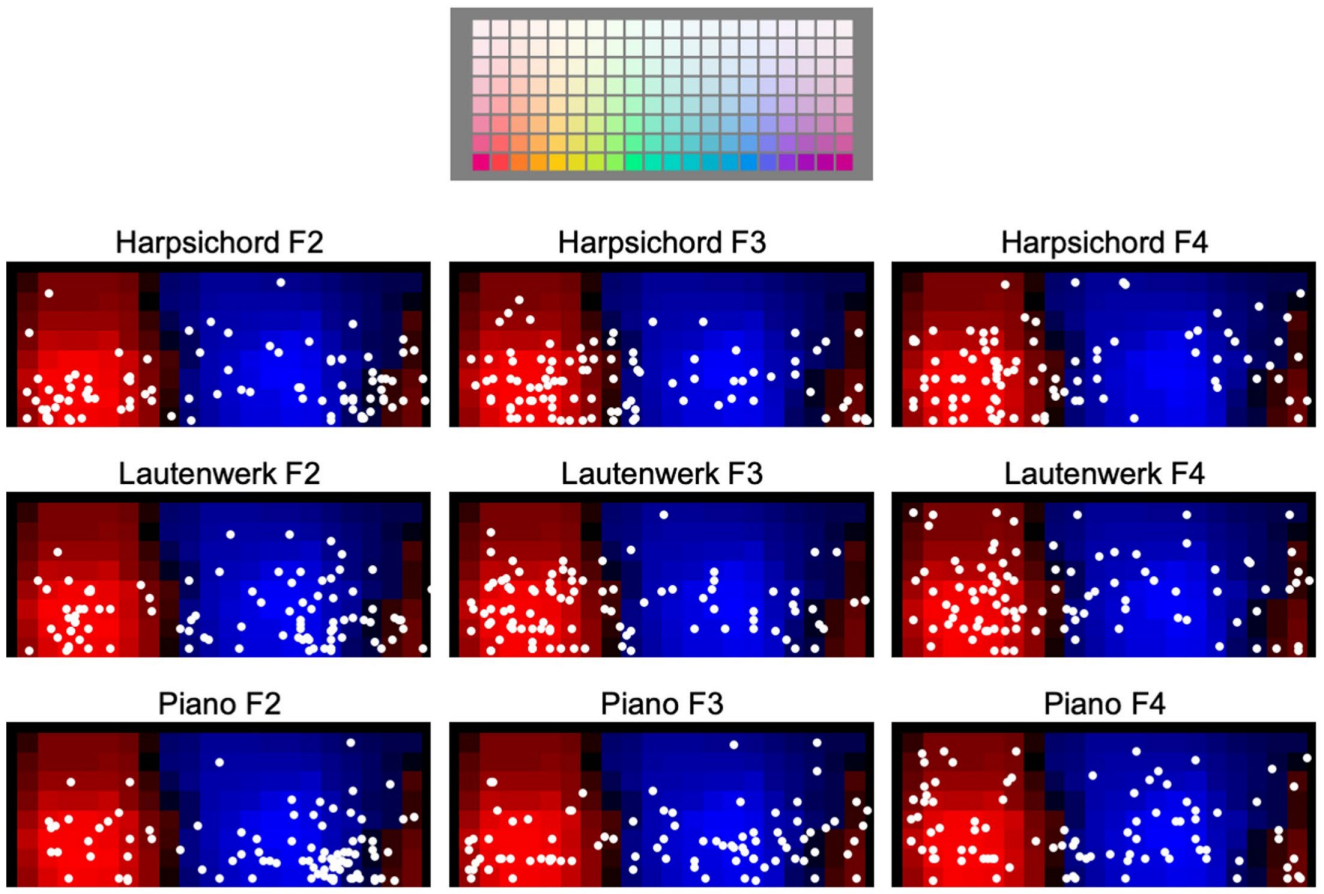
Centroids of participants’ color selections for keyboard instruments in Experiment A. Each white dot in each panel plots one centroid for one participant. Plotting convention is by hue vs. saturation, as depicted in reference panel at top of figure. The background shades of red and blue shown in the nine lower panels depict warm-cool consensus for the palette colors obtained in a separate control experiment (details in Supplementary material S1).

The panels in Figure 3 show the centroids (circular mean hue and mean saturation) for participants’ color selections for each instrument/pitch register condition in Experiment A, irrespective of luminance settings. Centroids are plotted on top of a background depicting the regions of warm (reddish) and cool (bluish) palette colors based on a template, derived from our control study (see Supplementary material S1). Circular means (see Mardia and Jupp, 2009) were computed as the mean angular location of the color selections on a color circle, assuming all 20 hues represented in our color palette are equally spaced on a circle. These angles were then mapped onto the horizontal dimension of each panel shown in Figure 3. The saturation component (vertical dimension) of each centroid was calculated as the arithmetic mean of saturations of the colors selected by a participant.

Note that while participants’ centroids exhibit considerable dispersion across the individual panels, there is a tendency in some panels, such as with the Lautenwerk at F2, for centroids to aggregate in multiple regions.

To operationalize the degree of warmth vs. coolness of color choices, we calculated a “warm-cool index,” *I^m^*, based on the results of the control study (see Supplementary material S1). This statistic quantifies, for each stimulus, the average bias in each participant’s color selections toward either warm (positive values) or cool (negative values) color selections.

The warm-cool index, *I^m^*, was used in statistical tests of H1c, which predicted that ratings of timbre on the terms *warm* and *cool* are associated with the warmth-coolness of matched colors. Two regression models were built predicting the warm-cool index from ratings of the terms *warm* and *cool*, with participant ID included as a random intercept. No significant effect of ratings on the terms *warm* or *cool* were observed on the warmth-coolness of the selected corresponding colors (see Supplementary material S4.7 for model summaries). Table 3 includes results, with *p-*values as well as marginal and conditional R2 values for *warm* and *cool* models.

**Table 3:** Results of linear mixed models predicting the warm-cool index of chosen colors from semantic ratings of *warm* and *cool*, including the *p-*values of the fixed effect along with marginal and conditional *R^2^* values.

Warm-Cool Index ~ Semantic Rating + (1 | Participant)						
	Experiment A (Keyboards)			Experiment B (Orchestral Instruments)		
	*p*	Marginal *R^2^*	Conditional *R^2^*	*p*	Marginal *R^2^*	Conditional *R^2^*
Warm	0.72	0.00	0.04	0.01	0.01	0.01
Cool	0.82	0.00	0.04	<0.001	0.02	0.02

Table includes results for both Experiments A and B. For individual model summaries and additional detail, see Supplementary material S4.7. Summary of models predicting warm-cool index from semantic ratings.

#### 3.3.2 Musical instrument (H2) and pitch register (H3)

To assess whether musical instrument (H2) and pitch register (H3) are related to color choices, we ran three two-way ANOVAs with lightness, saturation, and warmth-coolness as dependent variables. Independent variables for each model included pitch register (whether the scale began on F2, F3, or F4) and instrument type (piano, Flemish harpsichord, and Lautenwerk) as fixed effects, with participant ID as a random intercept (see Supplementary material S4.8 for further detail).

##### 3.3.2.1 Lightness

**Figure 4:**
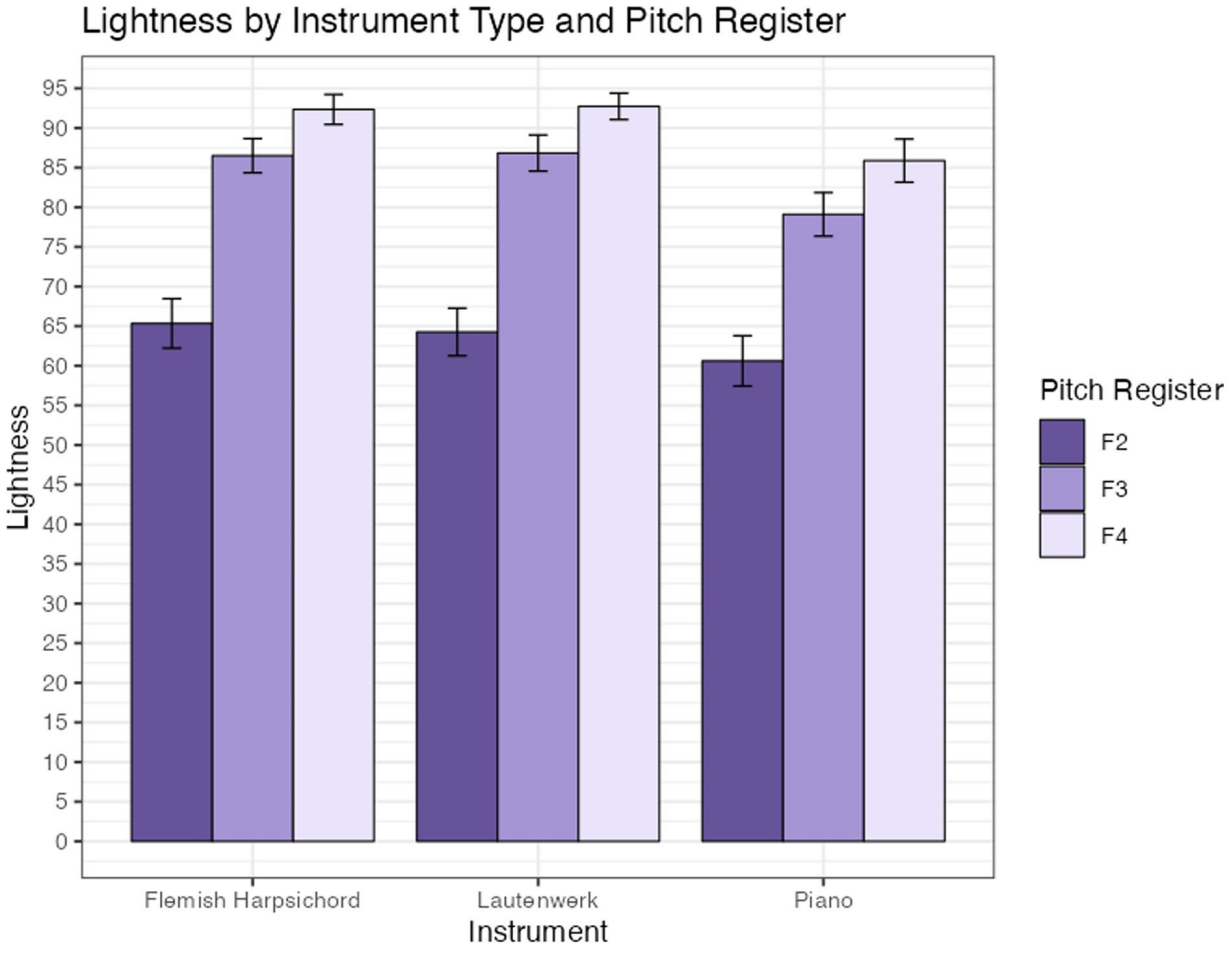
Mean color selection lightness for keyboard instruments across pitch registers in Experiment A. F2, F3, and F4 indicate the starting pitches for major scales. Error bars are +/− 1 SE.

A two-way ANOVA indicated differences in group lightness means for the three pitch registers [*F*(2, 568) = 131.18, *p* < 0.001] and for the three instrument types [*F*(2, 568) = 5.39, *p* = 0.004]; their interaction was not significant [*F*(4, 568) = 0.25, *p* = 0.91]. As with the semantic models, only observations from participants who used the slider more than once are included. Figure 4 shows lightness as a function of pitch register for each of the three keyboard instruments.

##### 3.3.2.2 Saturation

**Figure 5:**
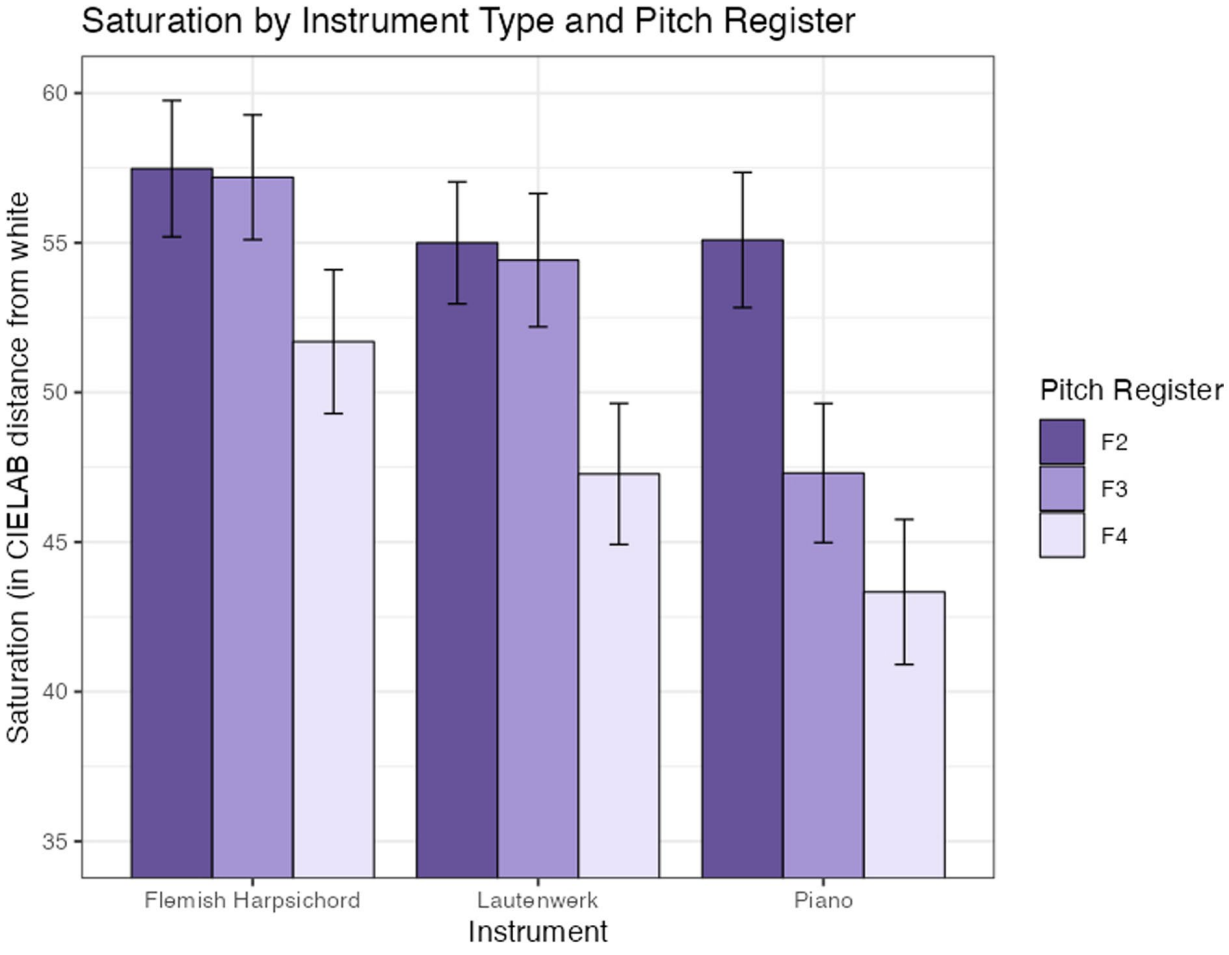
Saturation (in CIELAB units from white) for keyboard instruments across pitch registers. F2, F3, and F4 indicate the starting pitches for major scales. Error bars are +/− 1 SE.

A two-way ANOVA indicated differences in group means for the three pitch registers [*F*(2, 760) = 12.43, *p* < 0.001] and instrument [*F*(2, 760) = 8.03 *p* < 0.001], while their interaction was not significant [*F*(4, 760) = 1.13, *p* = 0.34]. Figure 5 plots saturation as a function of pitch register and instrument.

##### 3.3.2.3 Warm-cool

**Figure 6:**
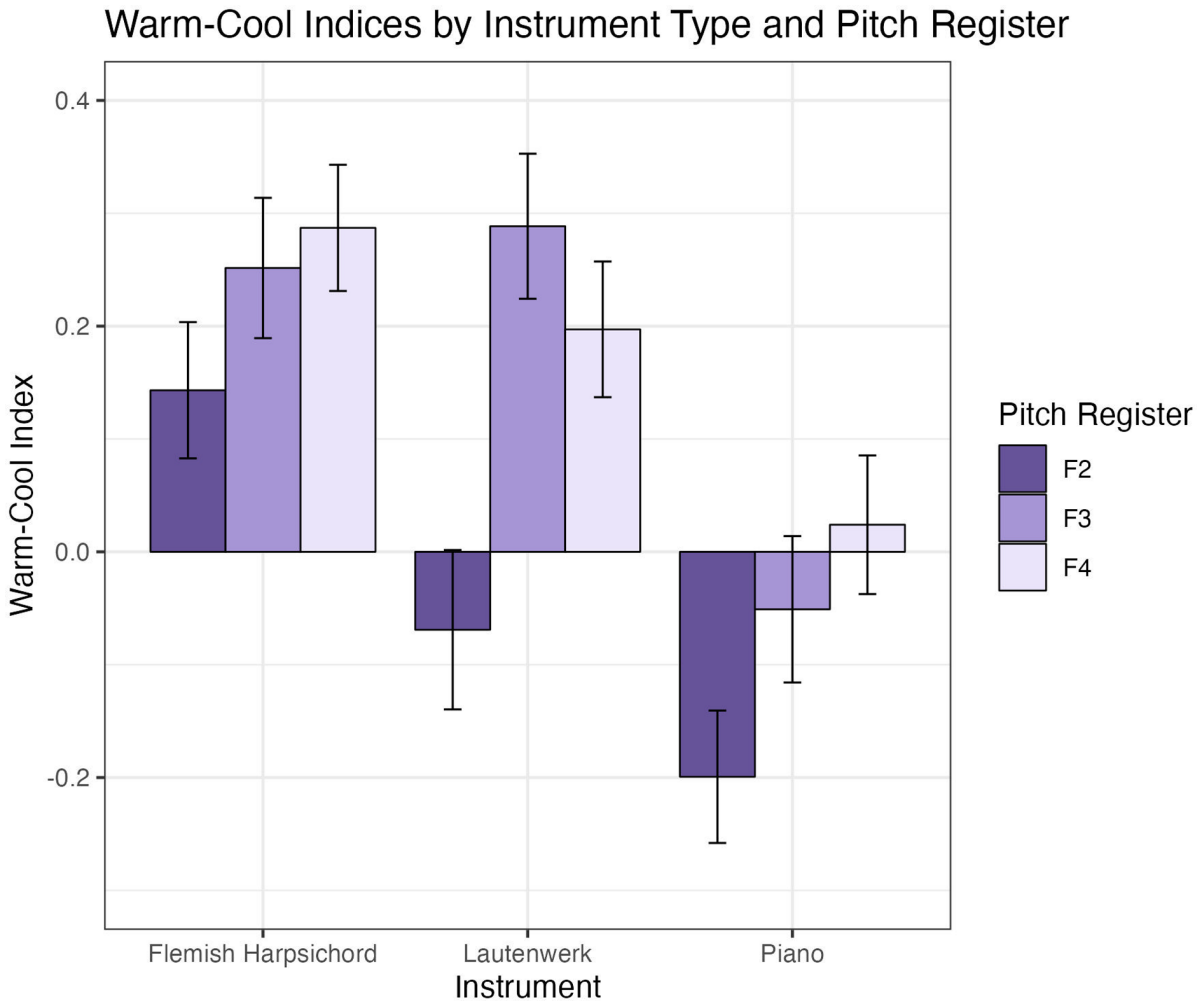
Mean warm-cool indices for each instrument/pitch register condition. Error bars are +/− 1 SE.

A two-way ANOVA identified significant effects of both instrument [*F*(2, 760) = 19.71; *p* < 0.001] and pitch [*F*(2, 760) = 11.74; *p* < 0.001] with no significant interaction [*F*(4, 760) = 1.33; *p* = 0.26]. Figure 6 illustrates mean warm-cool indices across pitch registers and instruments.

## 4 Experiment B

### 4.1 Methods

Experiment A varied both instrument timbre and pitch register; Experiment B was designed to experimentally control for pitch register while investigating timbre-color correspondences across a more diverse range of instrument timbres.

#### 4.1.1 Participants

Ninety-two participants (39 M, 52 F, 1 genderqueer) were recruited from the Center for Science and Industry (COSI; *n* = 70) and The Ohio State University music school subject pool (*n* = 22), which is composed of second-year music students enrolled in Aural Skills. The participant group in Experiment B does not overlap with that of Experiment A. Two participants reported synesthesia, including one color-sound associator; three participants indicated they were unsure as to whether they experienced synesthesia. We did not exclude data from these participants.

#### 4.1.2 Stimuli

##### 4.1.2.1 Recordings

Experiment B tested H1 and H2 with six orchestral instruments: flute, oboe, B♭ clarinet, trumpet, violin, and viola. This set of instruments was selected based on a pilot study in which 12 musician participants were asked to fill out an open-response questionnaire that listed each semantic descriptor of interest (e.g., *bright*, *dark*) and asked for a single musical instrument whose sound was most representative of that descriptor. Because the purpose of Experiment B was to experimentally control for pitch across a larger group of instrument types than in Experiment A, we selected instruments that were able to play the same scale in a comfortable, middle range. One concern was avoiding extremes in range, as timbres in the extreme high or low register of an instrument are often noticeably different from the middle register; thus, we selected instruments whose overall ranges were as similar as possible.

Stimuli were recorded in a studio by professional musicians. Recordings used an AKG 414 microphone, set to cardioid pattern, through an API 3124 preamp, and Pro Tools, with an SSL Delta-Link interface, and were normalized to-18dBFS. The sampling rate was 44.1 kHz/24. Stimuli for Experiment B included flute, oboe, B♭ clarinet, trumpet, violin, and viola each playing one-octave major scales from F4 to F5, approximately 10 s in length each.

##### 4.1.2.2 Color palette

The color palette was identical to that used in Experiment A.

#### 4.1.3 Procedure

Aside from the use of different instruments in the musical stimuli, the procedure was identical to Experiment A.

### 4.2 Results

**Figure 7:**
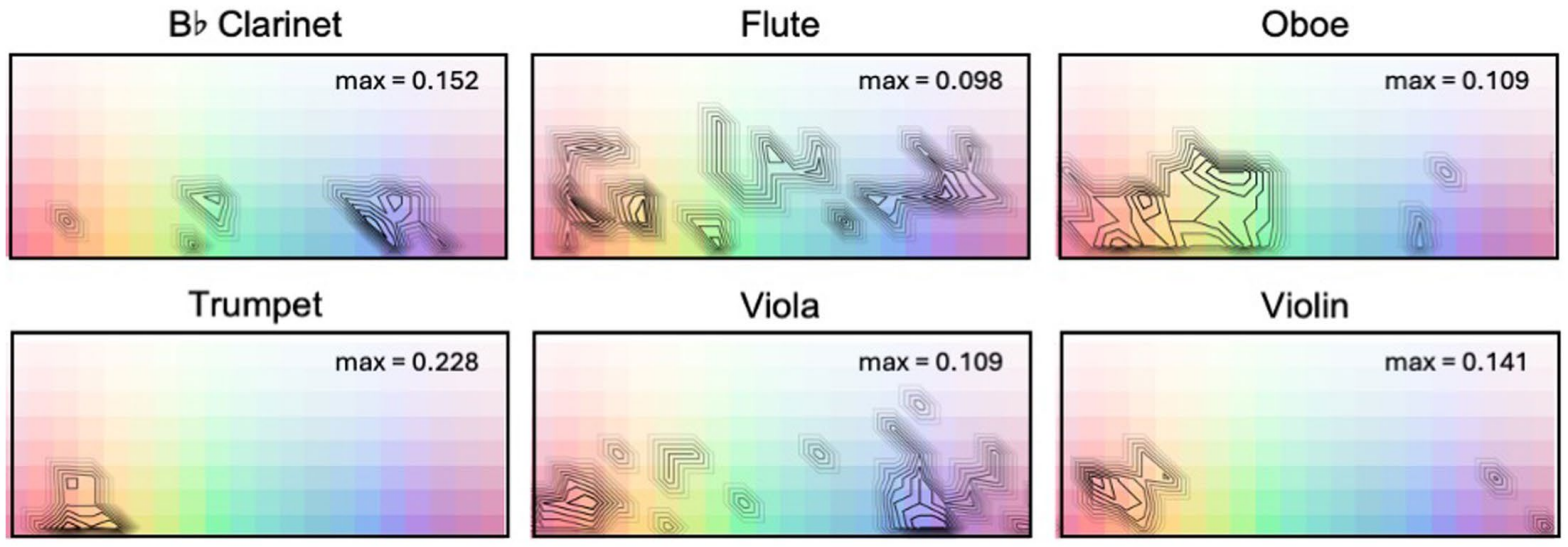
Contour plots of relative consensus in color selections (irrespective of luminance settings) for orchestral instruments studied in Experiment B. Maximum consensus for each instrument is indicated in the upper-right corner of each panel. Contours have been adjusted in contrast to highlight regions of color plots with highest consensus values.

Consensus in participants’ color selections for the six orchestral instruments tested in Experiment B are shown in Figure 7. The format of each panel is the same as that for Figure 2, above, where contours indicate areas of relatively high consensus, and consensus is calculated irrespective of participants’ luminance settings. Maximum consensus values are displayed in the upper-right corner of each palette. In Experiment B, the median number of color samples chosen was 4 (IQR = 4) with a minimum of 1 and a maximum of 49.

While it is apparent that there is diversity in color selection, it is also clear that there is more agreement for some instruments than others. The flute and viola do not demonstrate obvious hue bias, although green and blue responses for the viola are less common compared to other hues. The oboe spans a number of hues, but color selections are more concentrated than for the flute or viola, avoiding teals and purples. Color selections for the trumpet show a hue bias toward reds, oranges, yellows. The violin demonstrates a tendency to be matched to reds and oranges. Clarinet selections are somewhat concentrated on blues.

### 4.3 Analysis

Analysis for Experiment B replicates the procedures described for Experiment A.

#### 4.3.1 Semantic ratings

##### 4.3.1.1 Lightness

As in Experiment A, a subset of participants (*n* = 22) did not use the slider when choosing colors, defaulting to the lightest setting, while another subset of participants (*n* = 9) adjusted the lightness slider for only one of the stimuli, suggesting that they decided after the first trial not to adjust the lightness on subsequent trials. We report lightness models based on the subset of participants (*n* = 61) who made use of the slider for more than one trial.

Most models yielded a significant effect of semantic ratings on lightness of color selections, except for the terms *small* and *big*. Table 1 includes the results across the 10 linear mixed models predicting lightness; summaries of all parametric and nonparametric models are included in Supplementary material S4.4.

##### 4.3.1.2 Saturation

In relating semantic ratings to the saturation of matched colors, only the terms *light in weight* and *big* yielded significant results, with small effect sizes. A summary of saturation models for Experiment B is included in Table 2; summaries of all parametric and nonparametric models are included in Supplementary material S4.6.

##### 4.3.1.3 Warm-cool

**Figure 8:**
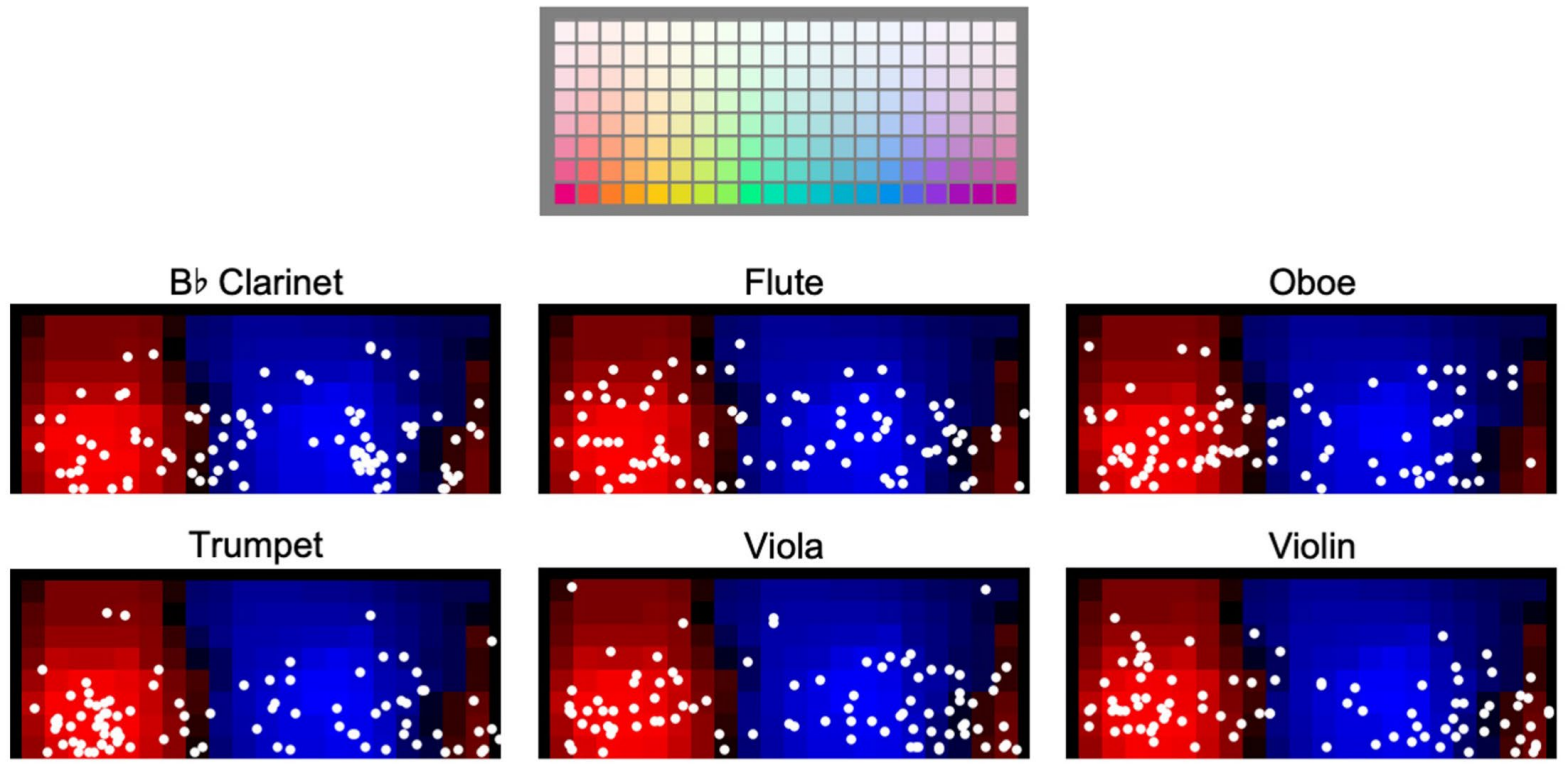
Centroids of participants’ color selections in Experiment B. Format of plots as in Figure 3.

Centroids for participants’ color selections for the six orchestral instruments tested in Experiment B are shown below in Figure 8. Background colors depict degrees of warmness (reddish areas) and coolness (blueish areas) associated with colors in the selection palette. Centroids are dispersed across both hue and saturation, particularly for the flute. As in Experiment A, some instruments (e.g., clarinet) show clusters in multiple regions.

As in Experiment A, two regression models were built predicting the warm-cool index, with semantic terms *warm* and *cool* each as a fixed effect and participant ID as a random intercept in both models. Both yielded significant fixed effects with low effect sizes (*warm*: *p* = 0.01, marginal *R^2^* = 0.01; *cool*: *p* < 0.001, marginal *R^2^* = 0.02; see Table 3). Fit for the linear mixed models for both *warm* and *cool* was singular due to zero variance for the random intercept. Refitting the models without a random intercept resulted in highly similar results that were in agreement on the significance of terms.

#### 4.3.2 Musical instrument (H2)

##### 4.3.2.1 Lightness

Experiment B experimentally controlled for pitch register, allowing evaluation of H2 across a more diverse set of six different orchestral instrument types. To test whether instrument type has an effect on lightness of color selections, we fit a linear mixed model including instrument as a fixed effect and participant as a random intercept, including observations only from those participants who used the slider more than once during the experiment. A one-way ANOVA indicated no difference in group means within the six instruments [*F*(5, 300) = 1.98, *p* = 0.08].

##### 4.3.2.2 Saturation

**Figure 9:**
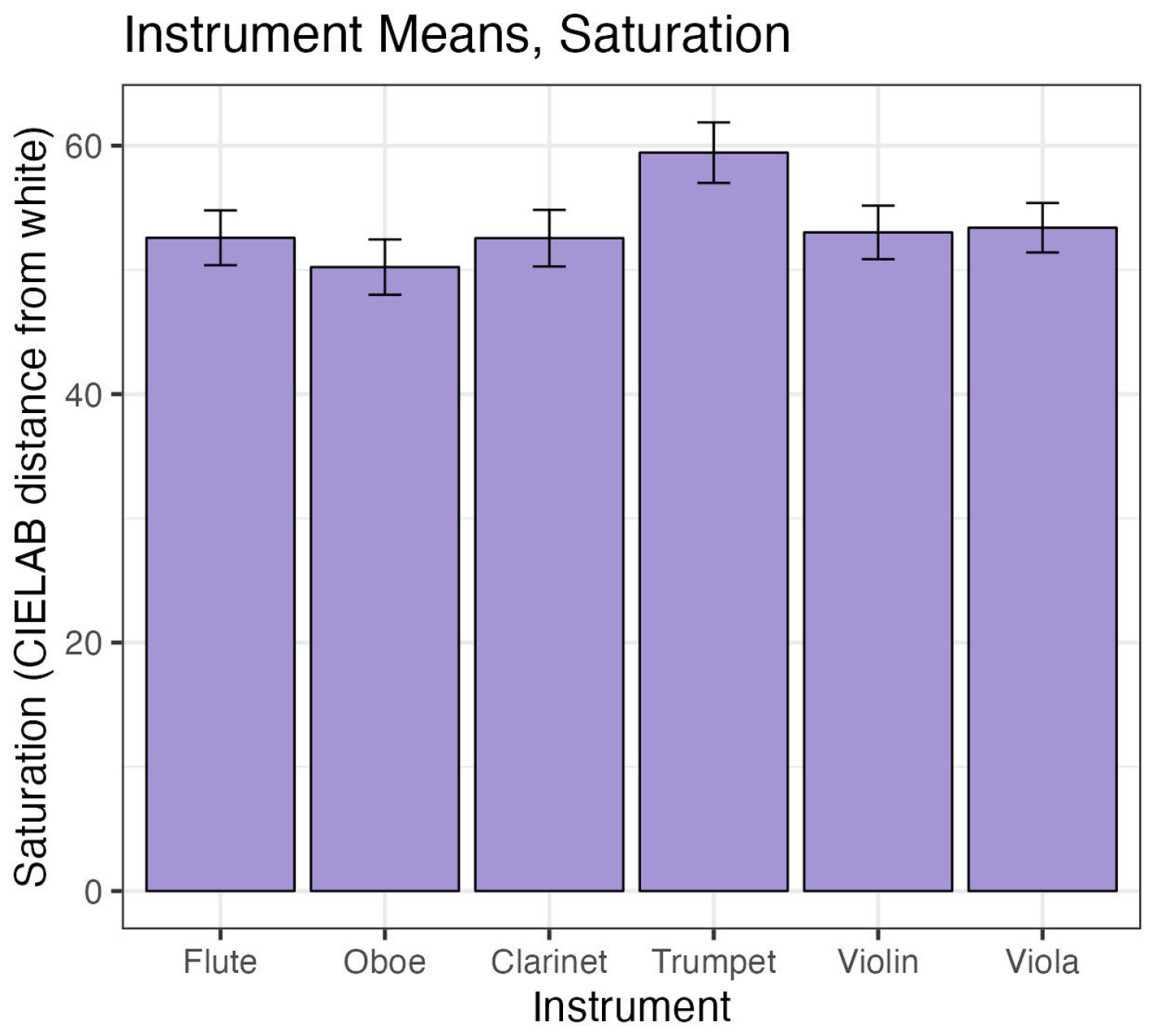
Average saturation (in CIELAB units from white) of color choices across participants for each instrument. Error bars are +/− 1 SE.

To test whether instrument type has an effect on saturation of color selections, independent of pitch, we fit a linear mixed model including instrument as a fixed effect and participant as a random effect, including observations from all participants. A one-way ANOVA indicated differences in group means within the six instruments [*F*(5, 455) = 2.89, *p* = 0.01]. However, this difference appears to be solely driven by the trumpet in comparison to the other five instruments, as suggested by the plot in Figure 9.

##### 4.3.2.3 Warm-cool

**Figure 10:**
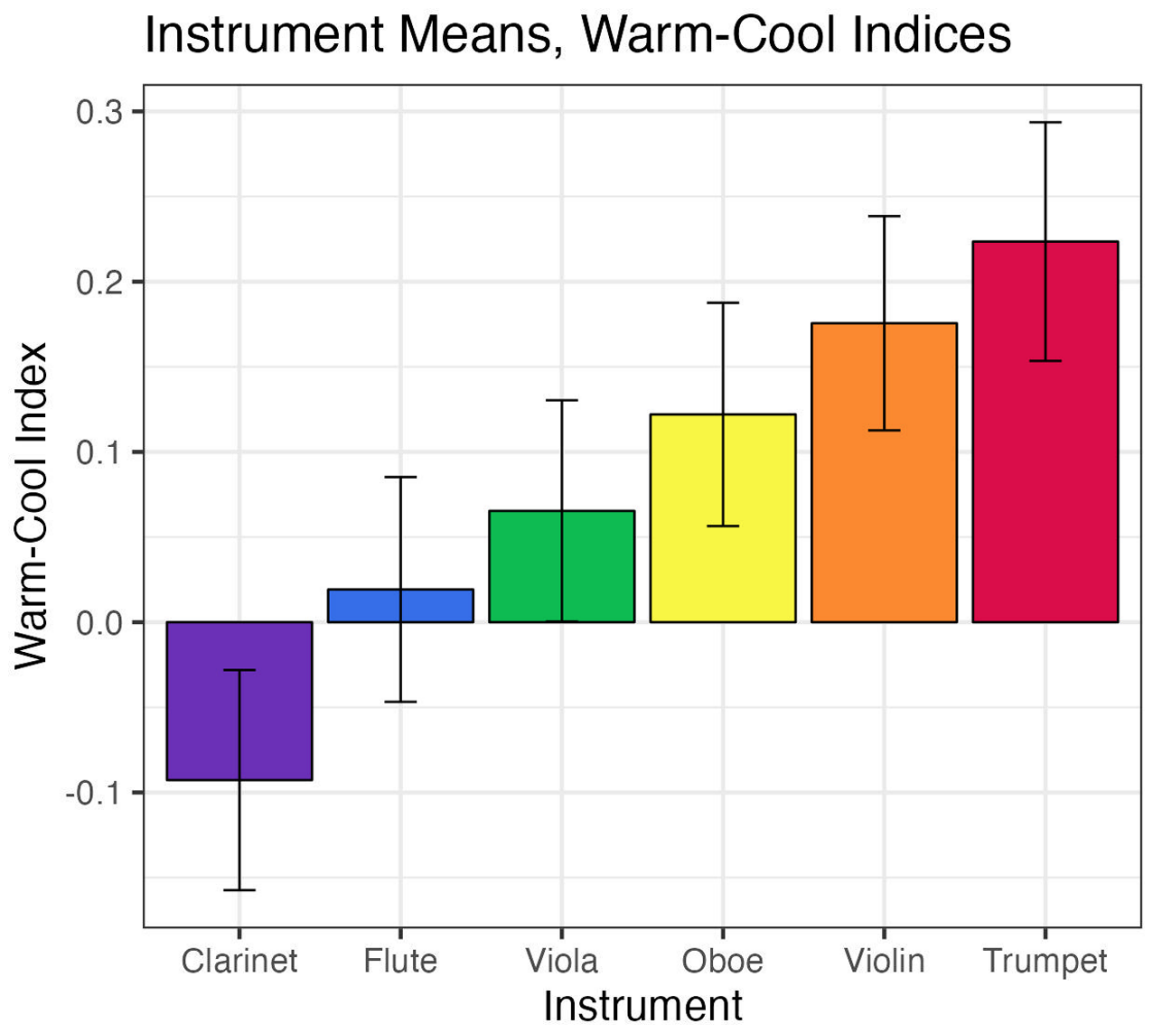
Average warm-cool indices by instrument, arranged in ascending warm-cool index order. Error bars are +/− 1 SE.

A one-way ANOVA revealed an overall effect of instrument type on warmth-coolness [*F*(5, 455) = 3.03, *p* = 0.01]. The bar chart in Figure 10 plots warm-cool indices *I^m^* for the test instruments, arranged in ascending index order from average cool (clarinet) to average warm (trumpet) color selections (see 3.3.1.3 for an explanation of *I^m^*). However, as was the case for the keyboard instruments tested in Experiment A, care must be taken in interpreting these results, given the substantial dispersion and, in some cases, bimodal aggregation, of the data.

## 5 General discussion

### 5.1 Semantic ratings (H1)

#### 5.1.1 Lightness (H1a)

Overall, results are consistent with H1a, which posited that semantic characterizations of timbre predict lightness of timbre-color matching. All terms were statistically significant in Experiment A, which systematically varied pitch register; in Experiment B, where pitch register was experimentally controlled, all terms except *small* and *big* were significant. Effect sizes for significant terms, as quantified with pseudo-*R^2^*, were larger in Experiment A, when pitch register varied (range = 0.08–0.16) than in Experiment B (range = 0.01–0.03), when pitch register was held constant.

Ratings on the semantic descriptors *small* and *big* were significant predictors of lightness when pitch register varied systematically, but not when it was held constant. Results of Experiment A are consistent with established understanding of sound-size symbolism, where higher pitch is perceived as corresponding to small objects and lower pitch is perceived as corresponding to bigger objects. That we did not observe the same relationship in Experiment B suggests that as descriptors of sound, these crossmodal terms are primarily or entirely driven by pitch register. Because we used ecologically relevant recordings of musical instruments as stimuli, we cannot fully disentangle change in fundamental frequency from timbral changes that correspond with increased pitch height. Because timbre varies with pitch height (Reymore, 2021; Reymore et al., 2023), crossmodal correspondences that vary with pitch register may be related to changes in both fundamental frequency and spectral components.

Yet, although *small* and *big* were not significant in Experiment B, the semantically related terms *heavy* and *light in weight* were. Among the eight significant terms in Experiment B, *light in weight* is ostensibly spurious, with a *p* value of 0.04. *Heavy* resulted in the lowest *p* value and highest marginal pseudo-*R^2^* among the Experiment B lightness models, but notably did not reach significance for the corresponding saturation model. Perhaps timbral weight carries more relevance for lightness, independent of pitch register, than does size, but these relationships need further study for confirmation and clarity.

#### 5.1.2 Saturation (H1b)

Given established connections among saturation, pitch, and heaviness, we constructed H1b using the same set of terms derived for the lightness hypothesis. Results demonstrated that these terms were, for the most part, significantly related to saturation in Experiment A but not in Experiment B. Experiment A yielded clear significant relationships between saturation and ratings on *bright*, *high*, *low*, *light in weight*, *heavy*, *big*, and *happy*. The terms *dark* and *small* were significant in ordinal models but not the linear mixed models; *sad* did not reach significance in either type of model. In Experiment B, only two out of the 10 terms were significant: *light in weight* and *big*. Overall, marginal *R^2^* values for the saturation models are much smaller than those describing the lightness models, suggesting that in timbre-color matching, crossmodal associations have greater influence on lightness than on saturation. In Experiment B, fewer terms reached significance, and effect sizes were smaller as compared to Experiment A, consistent with the idea that correspondences with saturation are for the most part dependent on pitch height. Perceptions of timbres as *big* or *light in weight* may relate to saturation of color choice across sound sources independently of pitch register, but the effects are small.

We based H1b, which predicted increased saturation with higher ratings on *low*, *dark*, *heavy*, etc., on previous work that suggested connections between saturation, heaviness, and pitch height (e.g., Alexander and Shansky, 1976). In our experiments, we found results to be consistent with our stated hypothesis. As noted in the Introduction, however, other research (Hamilton-Fletcher et al., 2017; Anikin and Johansson, 2019) would suggest the opposite correlation—that higher ratings on *low*, *dark*, *heavy*, etc. would align with decreased saturation. One possible reason for the discrepancies between studies could be that timbre-saturation correspondences interact with hue selection. For example, in Hamilton-Fletcher et al. (2017), higher pitch was associated with yellower hues: bright yellow is necessarily light and highly saturated, so it is possible that in this context, the specificities of this hue correspondence overshadowed a more general tendency to match weight and saturation. Then again, Anikin and Johansson (2019) did not observe the association between frequency and yellow, instead capturing a weak correlation between frequency and blue, but they did observe higher pitch and higher spectral centroid to correlate with more saturated colors. Thus, it is also plausible that timbre-saturation correspondences are influenced by the experimental paradigm and/or the most salient features of the available auditory stimuli set.

Experiment B allowed for consideration of whether the correspondence between heaviness and saturation holds when timbral heaviness is considered independently of pitch height. Here, results were mixed, where ratings on the term *heavy* were not significant, but those on *light in weight* were—although, with a *p-*value of 0.02, the *light in weight* result may be spurious. It is intriguing that the other term significantly predicting saturation in Experiment B was *big*. Size is often semantically related to weight; yet, *small* was not significant in Experiment A. It may be that stimuli with greater timbral variation could reveal clearer relationships among size, weight, and saturation, but it is also possible that any such relationships are relevant and meaningful only when pitch height is varied, and/or that our mixed results are due to noise in the data. For both lightness and saturation models, the size and weight descriptors yielded mixed results across both studies. To clarify potential nuances, more variance among timbres may be necessary, and a more constrained experimental design may be better suited to disentangle these particular relationships.

#### 5.1.3 Warmth-coolness (H1c)

Ratings on the terms *warm* and *cool* significantly predicted the warmth-coolness of color choices in Experiment B but not in Experiment A. This may be in part because Experiment B offered more types of instruments with a wider range of timbral characteristics than did Experiment A. Effect sizes in Experiment B were small; it is possible that including a wider and more diverse set of instrument timbres would yield a larger effect and could be explored in more depth in future studies. As discussed in more detail below in 5.4, there seems to be a meaningful difference between timbral warmth and warmth of color.

### 5.2 Effect of musical instrument (H2)

H2 proposed a relationship between musical instrument type and color choice, operationalizing color choice, as in H1, in three ways (lightness, saturation, warmth-coolness). Results linking instrument type and lightness were mixed: the relationship was significant in Experiment A but not in Experiment B. Both experiments demonstrated significant differences among instruments with respect to saturation and the warm-cool index.

In Experiment A, pairwise tests carried out via the *emmeans* function in the emmeans package (Lenth, 2023) and adjusted using the Tukey method pointed to significant differences between the Flemish harpsichord and piano as well as between the Lautenwerk and piano, but not between the two harpsichords, for all three dependent variables. Among the orchestral instruments in Experiment B, only clarinet/trumpet and clarinet/violin contrasts were significantly different for saturation (see Figure 10 for a visualization of all instruments’ mean warm-cool indices in Experiment B). With respect to the differences in warm-cool index, the trumpet appears to be solely responsible for the significant ANOVA result (see Figure 9). Taken together, it seems that there may be certain instruments that are characterized by relatively high or low values for one or more dimensions of color, such as the trumpet, but that these dimensions are not equally relevant for all instruments and/or may only be evident with particular types of timbral contrasts. The extent to which relationships between instrument type and individual dimensions of color are driven by, or interact with, the motivation to match an instrument to a certain hue (such as the trumpet, to red), calls for further clarification.

### 5.3 Effect of pitch register (H3)

H3, which proposed a relationship between pitch register and color, was tested in Experiment A only. We observed significant differences in lightness, saturation, and the warm-cool index as functions of pitch register (see Figures 4–6), and no interactions were found with instrument type. Replicating findings established in previous scholarship, increases in pitch register were associated with increasing lightness. Saturation decreased with increasing pitch register, consistent with our hypothesis but inconsistent with some previous findings in the literature (see 5.1.2 for a more in-depth discussion). The piano and Flemish harpsichord both showed a consistent increase of color warmth with pitch register, while the Lautenwerk’s matched colors were warmest in the middle register. *Post hoc* Tukey tests of the warm-cool index model show significant increases in warm-cool index between F2 and F3, and F2 and F4, but not between F3 and F4, likely due to the Lautenwerk’s divergent profile.

### 5.4 Warmth-coolness: further discussion

Although our Experiment A findings suggest a positive relationship between pitch register and warmth of color choice, trends in the semantic ratings across pitch register reveal further complexity: ratings of *warm* are highest in the middle register (F3) for each of the keyboard instruments, while ratings of *cool* tend to increase with pitch register! The latter observation is consistent with the results of work by Wang and Spence (2017), where participants matched the experience of drinking cold water with higher pitch, as compared to room temperature and hot water. However, it is notable that as timbre semantic ratings, *warm* and *cool* are not simple opposites when it comes to their relationships with pitch height.

Research on timbre semantics provides some insight into these relationships. Through interviews and an online survey with expert participants, Rosi et al. (2022) found that “a *bright* sound has most of the spectral energy in the high frequencies. It is often a high-pitched sound, with clarity, definition, and similarities with a metallic sound…A *warm* sound encloses substantial spectral energy in the low-mid frequencies. It is a rather low pitch sound…A warm sound is pleasant, enveloping, and rich” (480). That all three keyboards were rated as timbrally warmest in the middle register (rather than the lowest) suggests a kind of sweet spot for timbral warmth, rather than a simple linear relationship between pitch height and warmth. This middle register peak is not unexpected in light of findings from Reymore et al. (2023) and Reymore (2021) that other plausibly pleasant dimensions—*smooth/singing* and *watery/fluid*—show an inverted-U relationship with register. If *warm* timbres are associated with middle and lower registers, but color warmth is associated with higher pitch register, this could explain the null finding for H1c in Experiment A. Perhaps, participants’ gravitation toward warmer color choices in timbre-color matching is better explained by a combination of increased pitch and increased timbral brightness than by timbral warmth. In Experiment B, we noted that the instruments with higher warm-cool indices (oboe, violin, trumpet) seem to be brighter and more nasal than those with lower values (clarinet, flute, viola). Examination of average ratings on *bright* confirmed that this casual observation is consistent with participant ratings. Taken together, these *post hoc* observations suggest that timbral brightness may be more closely related to color-based warmth than is timbral warmth.

Thus, although we did find a significant relationship between ratings on *warm* and *cool* and warmth-coolness of color choice in Experiment B (but not Experiment A), results should be interpreted with caution. The significant effect may have been driven by other factors or may depend on the sample of instruments tested. Future work should assess judgments across a wider range of instruments to test generalizability and identify specificities that may interact with broader trends.

### 5.5 Emotion mediation and semantic mediation

Palmer et al. (2013) posited that if correlations between color and musical excerpts were mediated by common emotional associations, they would find analogous results when asking participants to choose colors most/least consistent with music and with any other set of stimuli strongly associated with the same emotional dimensions. Results from both subsequent experiments designed to test this were consistent with the hypothesis. Previous research shows that participants can make judgments on perceived emotion of timbres based on short tones (e.g., McAdams et al., 2017; Korsmit et al., 2023), suggesting that it is possible for perceptions of emotion to drive timbre-color matching.

Should shared emotion, mood, or affect provide the best account for timbre-color matching, as posited in the emotion-mediation account, we could anticipate in our experiments that the terms *happy* and *sad* would provide equivalent or better explanatory power than other descriptors. In our experiments, ratings on the terms *happy* and *sad* were significantly related to lightness in both experiments, but *sad* was not significant for saturation in either experiment. For lightness, in Experiment A, *happy* and *sad* resulted in slightly lower marginal *R^2^* values among the significant terms; in Experiment B, *R^2^* values were equivalent to those for other terms. That is, our emotion terms seem to be less related to color choice than other terms in our set.

On one hand, it may be that *happy* and *sad* were not apt emotional descriptors for the available instrumental timbres, but that other emotional terms would have provided a better fit. However, this seems unlikely for the given musical context; Korsmit et al. (2023) found that two dimensions are sufficient for capturing variance in emotional assessment of single-note and chromatic scale stimuli. On the other hand, it seems likely that emotional qualities of isolated timbres are not always the most relevant consideration for a given task. Other, lower-level perceptual features might be more immediately relevant for timbre-color mapping. Indeed, Spence, (2020a) observes that emotion mediation tends to account for more variance for complex stimuli as compared to simpler and less emotionally valent stimuli. Similarly, from results of an auditory-conceptual association study, Di Stefano et al. (2024) note that their findings support the view that complex stimuli are more likely to generate emotional meaning than are simpler stimuli (e.g., isolated sounds). Future research could test the emotion mediation hypothesis using Palmer et al.’s (2013) method with isolated timbres to directly address the claim that this explanation is equally applicable to complex and simple stimuli.

Spence (2011) proposed three categories motivating crossmodal matching, including semantic, physiological, and statistical, where semantic mediation relates to linguistic or lexical correspondence (e.g., we use *high* to describe both elevation and pitch). Motoki et al. (2023) added the category of “affective” to this model to account for emotion mediation (see also Spence, 2020a). These categories are not mutually exclusive, and a given correspondence may be motivated by multiple categories. As we chose our terms for this study based on crossmodal terms observed in previous timbre semantics research, it seems reasonable that semantic mediation provides an overall better explanation for our results, at least with respect to lightness and saturation. Notably, the terms *happy* and *sad* are used less often to describe timbre as compared to the other terms in our set—from a semantic mediation perspective, this may explain why these terms explained relatively less variance.

### 5.6 Hue

Participant color choices for each instrument were diverse. Some instruments demonstrated no specific trends in hue but did show heavier concentrations of responses at particular lightness levels, such as the flute. Some instruments demonstrated trends in hue, such as the trumpet, violin, and clarinet. While specific instrument-hue associations from our participants only seem apparent for a few instruments, the origins of these associations provide an intriguing subject for further research, particularly in the case of the trumpet, which has shown robust hue correlations in both historical and empirical studies (Reuter et al., 2018).

In the case of the keyboard instruments, pitch register appears to have played an important role: the lowest octave showed a concentration on saturated blues and purples for all three instruments. The piano was associated with a dispersed range of hues in the top two octaves, but both harpsichords showed an increasing tendency toward yellows as pitch increased. For the harpsichords at F2 in Experiment A, we speculate that participants may have been responding to two different aspects of the sound—some may have chosen colors primarily on the basis of pitch register (blues and purples), whereas another group may have been influenced by the brighter timbres of the harpsichord to select oranges and reds, explaining the bimodal concentration of responses in Figure 2. In general, color choices for the two types of harpsichords are far more similar to each other in each octave than they are to the piano and are generally associated with warmer colors as compared to the piano. Similarities in color choices between the two types of harpsichords may reflect their relative perceived similarity in timbre as compared to the piano.

### 5.7 Limitations and considerations for future studies

One limitation of our approach to analysis was our use of the mean in quantifying the saturation and warmth-coolness of participant color choices. Participants often selected multiple colors for a stimulus; for such observations, the saturation and warm-cool values used in modeling were approximated using the mean. This becomes potentially problematic when participants selected colors in different areas of the arrays. For example, if a participant selected a low saturation color and a high saturation color, the average saturation of their response is in the middle, which may not be representative of their response tactic. However, before settling on the use of the mean for analysis, we manually reviewed the data and determined that color selections were usually made in single clusters of contiguous color samples, for which the mean was a reasonable representation.

As previously mentioned, participants were not required to use the slider during their selections, and the data reveal that a subset of participants did not use the slider when choosing colors, defaulting to the lightest setting. It is unclear whether participants were intentional about this and felt that the lightest palette best exemplified the colors they wanted, misunderstood the directions, or opted not to use the sliders in order to get through the experiment more quickly. In experiments using a similar interface, we recommend enforcing slider use by requiring participants to acknowledge the slider via touch, even when they prefer to leave it in its initial location. We also acknowledge the possibility that for some subjects and conditions, even our most luminous palettes might not have been sufficiently luminous to match the subject’s subjective appraisal of the musical recording.

It should be noted while the use of the major scale for stimuli facilitated comparisons among instruments and allowed us to directly compare semantic ratings and color choices, using stimuli with different musical parameters (mode, articulation, rhythm, etc.) would likely result in somewhat different choices in both semantic descriptions and colors. While we hypothesize that the relationship between semantic descriptions and colors would hold in other simple musical contexts, future research might vary musical parameters to test the generalizability of our results.

Finally, though some portions of the variance in lightness and saturation were explained from the semantic ratings, the majority of variance in each case remains unexplained; a number of other factors likely influence timbre/color matching behavior. It should be noted that the purpose of the experiments reported here was not to thoroughly model the timbre-color matching process, but rather to test whether crossmodal language is related to timbre-color associations. Our results provide converging evidence in support of this theory but also suggest that the issue is multi-layered and complex, and that there are likely multiple principles guiding timbre-color matching behavior.

For example, interactions among the three color dimensions, particularly between hue and each of the other dimensions, may account for some of the variance in color choice. The trumpet provides a likely example of this. Color selections for the trumpet appear to be hue-focused: selections near red, orange, and yellow were most often selected. If a participant hears the trumpet and immediately thinks of a basic color category, such as that exemplified by a typical fire-engine red, the imagined hue necessitates a particular level of lightness—the lightness of the choice is in some way an artifact of the hue. Similarly, if a participant associates a sound with a typical yellow, the selection will be necessarily on the higher end of the lightness scale because as yellow darkens, it becomes brown. However, Hamilton-Fletcher et al. (2017) previously found crossmodal associations between non-musical sounds and color, including a relationship between yellow and high frequencies, even when controlling for the influence of lightness. Such observations raise the possibility that similar types of relationships may have been at play in the current study concerning instrumental timbre (note specifically the hue results among the keyboard instruments), though further research is needed to determine whether that is the case for the types of stimuli studied here and how such relationships may be connected to participants’ use of crossmodal language.

Although we did not observe that ratings on *happy* or *sad* were privileged among semantic terms in predicting color, timbre-color matching might be more generally a product of valence transfer (e.g., Weinreich and Gollwitzer, 2016). Emotion might also influence color choice via participant mood. For example, perhaps participants in a happier mood might be generally more prone to choosing lighter or yellower colors. Another potential mediating variable could be personal preference. Participants may be more likely to choose colors that they like, but preference might also play a more complex role: for example, participants might match colors they like to timbres they like and colors they dislike to timbres they dislike, where preference interacts with valence or qualia transfer.

Additionally, some color matching choices for recognizable instruments may be primarily driven by semantic rather than perceptual associations derived from cultural tropes. This might be the case for the trumpet’s association with red or the clarinet’s association with blue. Even if this is sometimes the case, however, it may still be possible that some of these cultural associations have perceptual origins, and so the two cannot be completely separated without further investigation. Future work could assess participants’ familiarity with instruments, which may help identify such connections.

We did not observe any particular patterns or differences among participants with sound-related synesthesia, though there were only a few such participants. The question of how synesthesia might interact with timbre-color associations could be addressed in a subsequent study with a larger population of synesthetes. In general, further systematic investigation of effects of other individual or cultural differences could also help contextualize variability of responses.

Finally, although the number of stimuli in the current experiment limited the generalizability of audio feature analysis, future research might include a larger and more varied stimulus set to assess the impact of particular features, such as spectral centroid. A stimulus set with increased timbral diversity would help clarify such potential relationships and might reveal more pronounced differences in semantic ratings and/or color choices. Future work might expand beyond instruments in the Western orchestral tradition to include instruments from other musical traditions in order to widen the array of available timbres.

## 6 Conclusion

Implicit in our experimental design and those guiding other studies is the assumption that the three putative perceptual dimensions of color—hue, saturation and lightness (brightness)—are separable (see Garner, 1974) and that we can probe for timbre/color correspondences related to each dimension independent of the other two. However, our results suggest that these correspondences may arise from more holistic mental representations of color and timbre. Though we find lightness is most closely associated with variations in timbre, there are clear interactions among the different dimensions of color that are evident in the data and complicate this picture. Moreover, for some experimental conditions, participants’ color selections tend to cluster in a few regions of our color palette. This suggests that for these conditions at least, individual differences in timbre-color correspondences are not entirely random but are guided by a few distinct mental representations of timbre-color correspondence.

Taken together, our findings demonstrate that crossmodal timbre semantic terms bear relation to timbre-color matching behavior, particularly in relation to the lightness of selected color samples. These results are consistent with semantic mediation (Spence, 2011) for color-timbre correspondences. Effects are larger when both pitch register and sound source are varied, but they are observable even when pitch register is held constant. The 10 terms proposed in H1a and H1b are more robustly related to the lightness of selected colors than to saturation. With respect to saturation, only two terms reached significance when pitch height was controlled. We found a weak relationship between ratings on *warm* and *cool* with the warmth-coolness of matched colors among orchestral instruments in Experiment B, but not among the keyboard instruments in Experiment A. Overall, evidence is consistent with H2, which posited that color choice varies among musical instrument types, but that these relationships are complex and involve specificities. Lightness differences were found in Experiment A but not B, while saturation and warm-cool differences were found in both experiments. However, *post hoc* comparisons suggest that these significant results were typically motivated by contrasts between particular instruments. Finally, results supported H3, which predicted a relationship between pitch height and color. Significant differences across pitch registers were found with respect to lightness, saturation, and the warm-cool index.

Diverse music have diverse musical goals and consequently prioritize different musical parameters. The relative salience of various parameters is important in determining which aspects of the music provide listeners the strongest cues related to crossmodal associations. There remains work to be done on understanding the relationship between crossmodal associations with basic sensory features and the emergent emotional interpretation that seems to play such an important role in crossmodal associations with composed music. The major scales used in these experiments represent a middleground: they are more complex than single notes but less complex than composed music, introducing a dimension of musicality while maintaining control of musical content. Previous research on crossmodal associations with basic sensory features has focused heavily on pitch; in order to relate crossmodal associations with simple stimuli to associations with complex stimuli, further research is called for on parameters other than pitch, such as timbre.

An understanding of common trends in timbre-color associations is especially relevant for the field of music visualization, in which visual images, colors, and shapes are set or co-created with music, with possibilities for artists working in musical multimedia continuing to grow as technology advances. The current consideration of instrumental timbre as simpler stimuli, outside of the context of more complex musical stimuli, may be especially relevant for those contemporary composers who approach timbre as critical for or central to their compositional process. In general, composers often seek to affect their audiences through understanding preferences and manipulating expectations; thus, a theory of audiovisual art necessitates a thorough exploration and understanding of crossmodal preferences and expectations, which likely relate to both the experience and aesthetic appraisal of multimedia art.

This is the first study to establish the conceptual relationships between the crossmodal linguistics of timbre and crossmodal correspondences with all three dimensions of color, and it is the first to address the interaction and relative contributions of timbre and pitch register to color-matching behavior with a range of musical instrument timbres. Our use of multi-note stimuli controlled for musical content adds a dimension of ecological validity to our results as they relate to topics such as the analysis of multimedia art, and the color palette offers colorimetrically precise three-dimensional variation in color and an expansive array of color choices to participants that far exceeds choices presented to participants in many music-color studies, while allowing for multiple color selection.

## Data Availability

The raw data supporting the conclusions of this article will be made available by the authors, without undue reservation.
