# Supplementary material for: Color and tone color: audiovisual crossmodal correspondences with musical instrument timbre
Source: Front Psychol. 2025 Jan 7;15:1520131. doi: 10.3389/fpsyg.2024.1520131 (PMC11747214; doi:10.3389/fpsyg.2024.1520131)
Supplement: Supplementary file 1 [file Data_Sheet_1.PDF]

Supplementary Materials

S1. Warm-Cool Control Study

In Experiments A and B described in the main text, we computed warm-cool indices for our participants' color selections for musical instruments. These indices indicate the degree to which a particular test color should be considered "warm" (positive values) or "cool" (negative values),(see Equ. S1, below) by a typical participant. These indices were derived from measurements of consensus in the assignment of each of our 160 test colors (20 hues x 8 saturation levels; see Figure 1 of main text) to either a "warm" or a "cool" color category (see Holmes, et al., 2017, for another recent study of the warm-cool color classification based on color samples used in the World Color Survey (Kay, et al., 2010).

S1.1. Stimuli, Control Study

Figure S1, below, recapitulates the stimulus array for our experiments shown in Fig. 1 of the main text. The palette colors, which averaged 258 cd/m^2 in luminance, were spatially arranged in a 20 x 8 palette of color samples organized according to hue (palette columns spanning the color circle) and saturation from most saturated (lowest row) to almost white (top row). These were displayed on top of an 85 cd/m^2 5000 Kelvin gray background. Also included in each display were black, white, and gray samples located in a row immediately above the color palette.

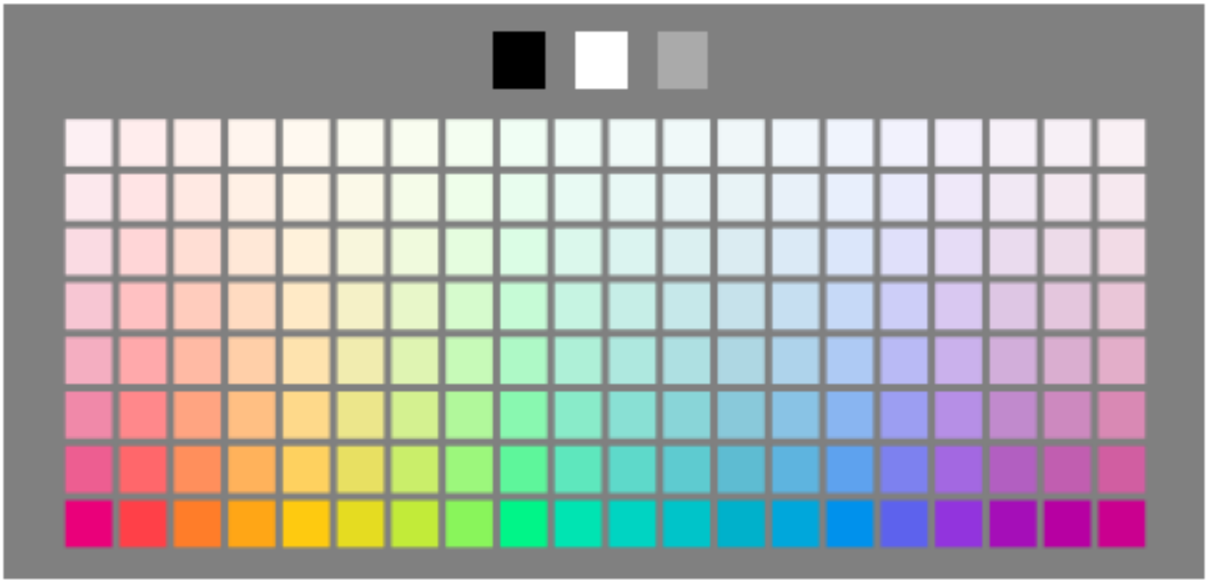

Figure S1. Color palette used in the warm-cool control study and in Experiments A and B, shown at the maximum luminance.

S1.2 Participants & Methods, Control Study

Thirty-five participants (14 male, M, 21 female, F) were recruited from the Ohio State University Student Union and ranged in age from 18 to 63 years (M = 26.6, SD = 11.5). Participants were tested on a colorimetrically calibrated (Photo Research PR-750, Chatsworth, CA, USA) iPad running a custom-programmed app. Participants viewed the palette at each of four different luminance levels: 0.2, 0.4, 0.7 and 1.0 times the maximum mean luminance of 264 cd/m^2. Each

sample's CIE {x,y} chromaticity coordinates were held constant across these four test luminance levels.

An experimental session in our warm/cool study consisted of eight trials, each corresponding to one of four color palette luminance levels, as described above, and one of two instructional conditions: to select either warm or cool colors. These eight luminance/selection conditions were tested in random order. On each trial, subjects selected all the colored elements in the display that, in their judgment, qualified as "warm" or "cool" colors (depending on the condition), and then pressed a button to register their color selections and advance to the next luminance/selection condition. In order to maximize the independence in participants' color selections across trials, participants were presented with a version of the palette that preserved the overall configuration of the one shown in Figure S1, except that, on each trial, the palette columns were randomly circularly rotated around the color circle and/or the palette rows reversed in order, so that on some trials saturation increased from top to bottom, while in other trials saturation increased in the reverse row order.

S1.3. Results, Control Study

Figure 5 shows consensus plots for cool (middle row) and warm (bottom row) selections by our 35 participants for relative luminances of 0.2 through 1.0. These panels were obtained by multiplying each stimulus palette color by the fraction of subjects who included that color among their warm or cool selections for a given luminance level. As expected, the ranges of warm selections span red, orange and yellow samples while cool selections span primarily greens, blues and, to a lesser extent, purples. There is a slight bias in both warm and cool selections towards high- and mid-saturation hues.

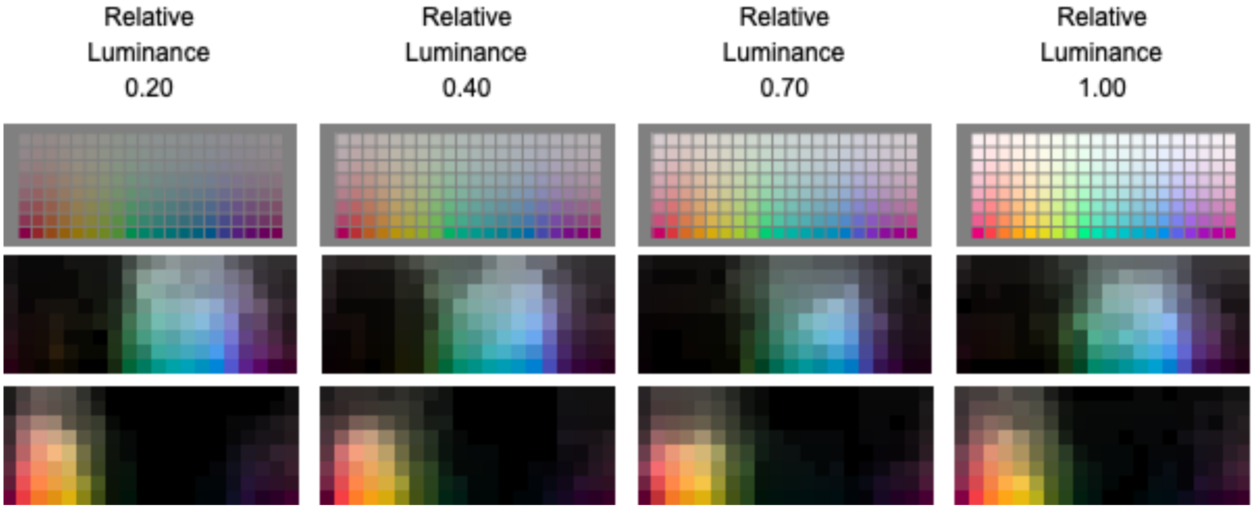

Figure S2. Consensus plots of warm and cool color selections as functions of mean palette luminance. Top row: Depictions of color palettes at indicated fractions (0.2, 0.4, 0.7, 1.0) of maximum mean luminance (264 cd/m^2). Middle row: Cool color selections for the four mean luminance test conditions. Here, as well as in bottom row, the brightness of each square check indicates degree of relative consensus for the corresponding color sample. Bottom row: Warm color consensus for the four mean luminance test conditions. Not shown: results for black, white and gray samples, which were rarely selected.

Participants' warm and cool color selections, shown separately in Fig. S2, were combined to create 160-element warm/cool templates, WC^k, one for each of the four mean luminances tested. The elements of each template, $WC^k_{i,j}$ (i = 1…20 hues, j = 1…8 saturation levels, k = 1…4 mean luminances), corresponded to differences in warm, $W^k_{i,j}$, vs. cool , $C^k_{i,j}$, consensus for the i,j^{th} color in the stimulus array under the kth $(k = 1\ldots4)$ test luminance:

$$WC^k_{i,j} = W^k_{i,j} - C^k_{i,j} \qquad \text{(Equ. S1)}$$

We define $WC^k_{i,j}$ as the warm-cool index for sample i,j and relative luminance k. Warm-cool indices for ensembles, Q, of color selections at a luminance setting L, correspond to ensemble averages of the individual sample warm-cool indices:

$$WC^L_Q = \sum_Q WC^L_q \Big/ N_Q \qquad \text{(Equ. S2)}$$

where N_Q equals the number of color selections in Q. Note that when L is not one of the test luminances, WC^L_q was obtained by linear interpolation between sample indices for q derived from Equ. S1.

Although we use linear interpolation in calculating warm-cool indices, inspection of Fig. S2 shows that warm and cool consensus, and hence $WC^k_{i,j}$, does not differ markedly across our K test luminances. Thus, for purposes of illustration in the warm/cool figures in the main text, a single warm/cool consensus plot was created, based on averages of warm and cool consensus across all test luminances.

References cited.

Holmes, K. J., & Regier, T. (2017). Categorical perception beyond the basic level: The case of warm and cool colors. *Cognitive Science*, *41*(4), 1135--1147. https://doi.org/10.1111/cogs.12393

Kay, P., Berlin, B., Maffi, L., Merrifield, W. R., & Cook, R. (2010). *The world color survey.* CSLI Publications.

S2. Quantile-Quantile (QQ) Plots and Histograms of Residuals of Model Fits to Semantic Ratings in Experiment A (Keyboards).

S2.1. Lightness *vs. X*

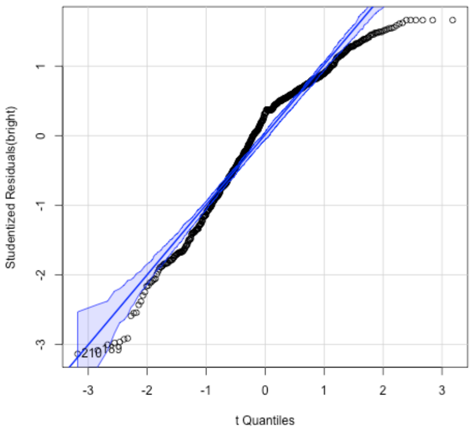

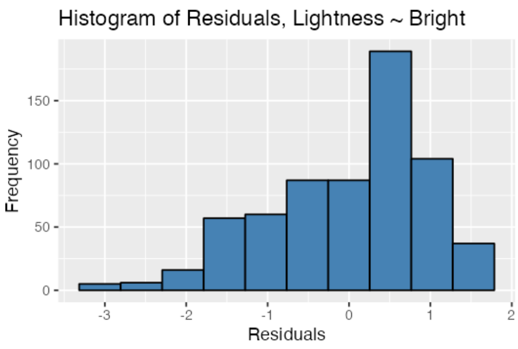

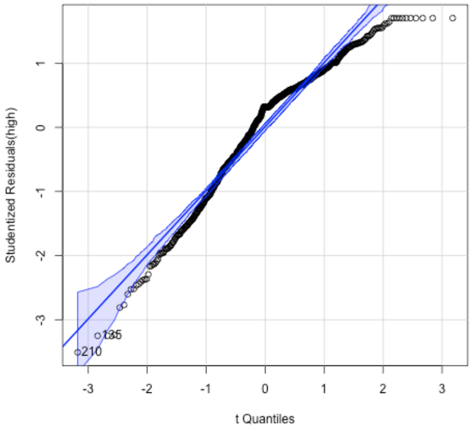

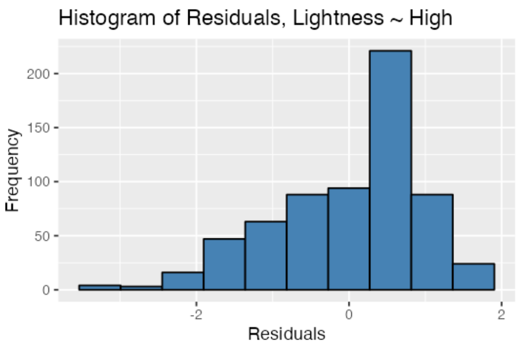

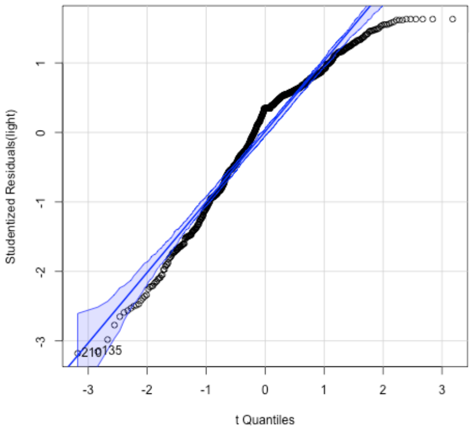

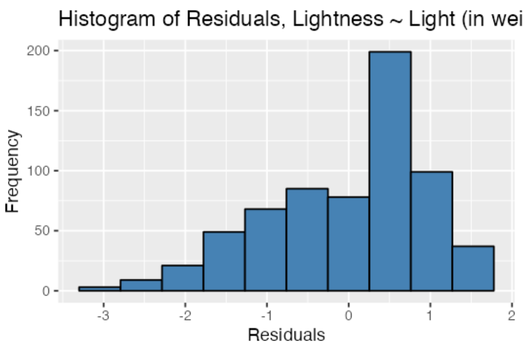

S2.1. QQ Plots and Histograms Experiment A cont'd: Lightness *vs. X:*

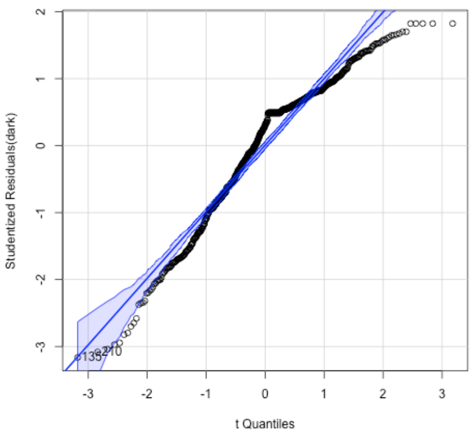

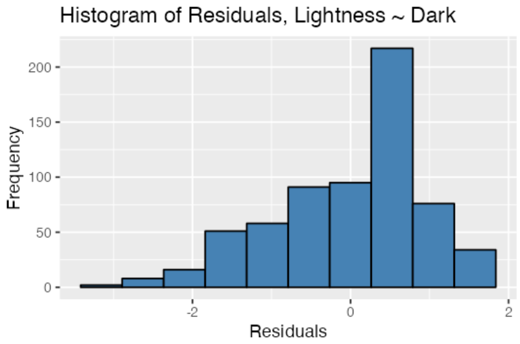

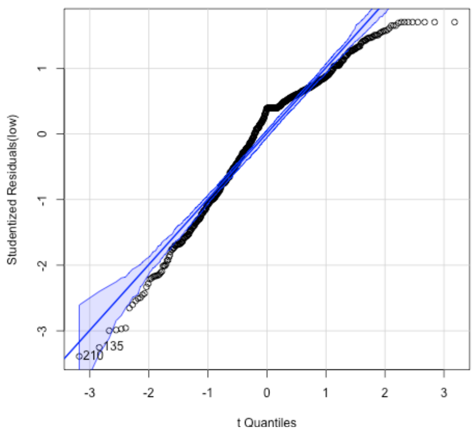

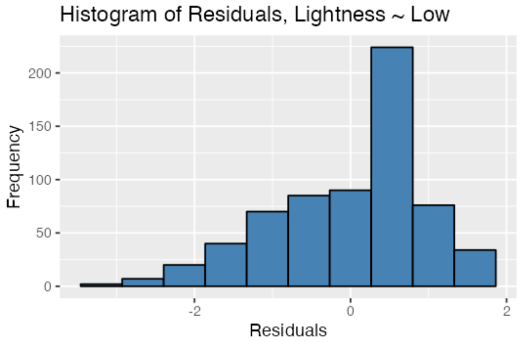

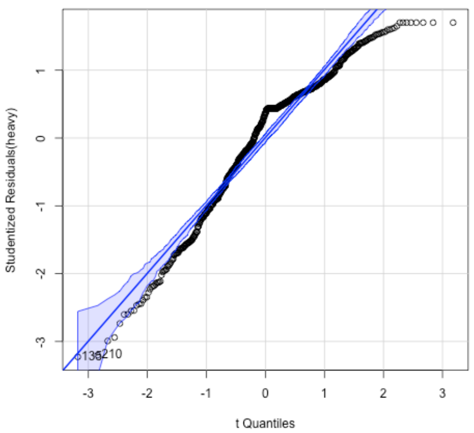

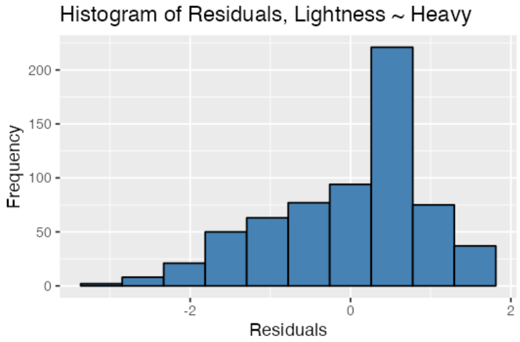

S2.1. QQ Plots and Histograms Experiment A cont'd: Lightness *vs. X:*

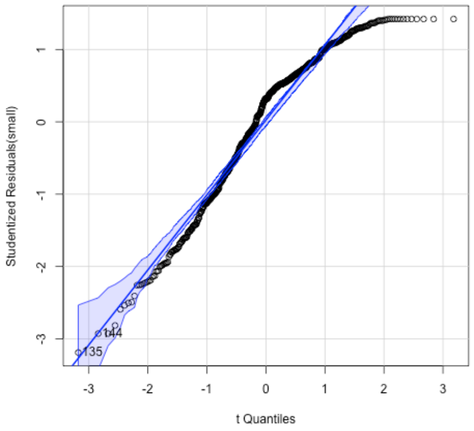

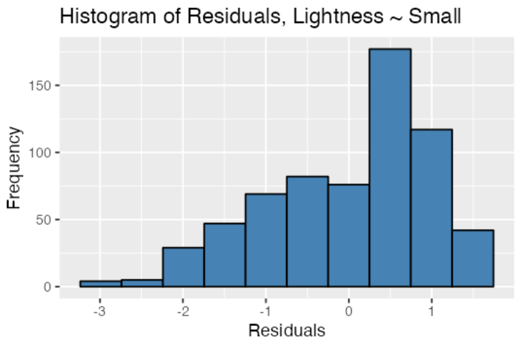

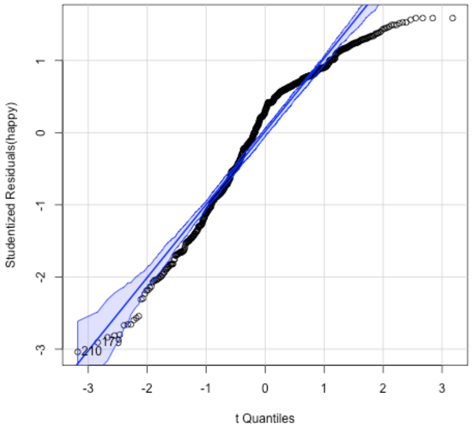

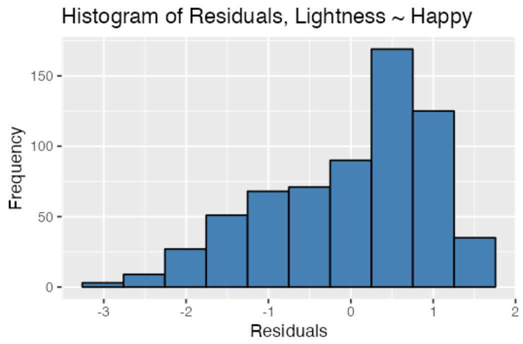

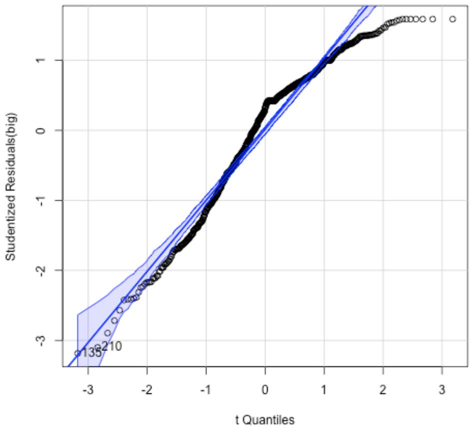

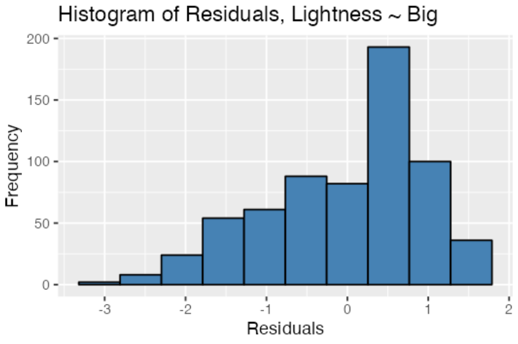

S2.1. QQ Plots and Histograms Experiment A cont'd: Lightness *vs.* *X:*

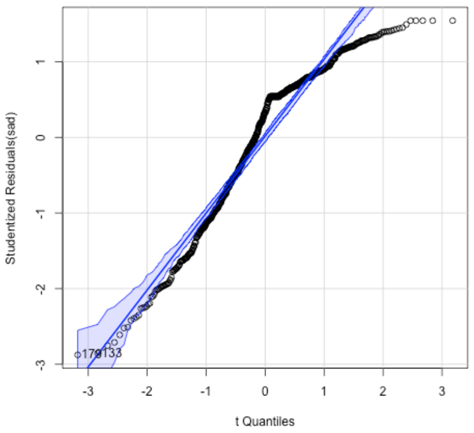

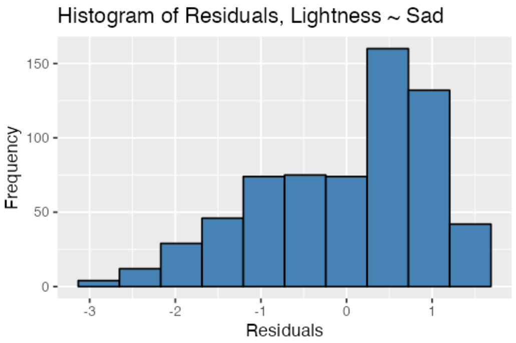

S2.2 . QQ Plots and Histograms: Experiment A Saturation *vs. X:*

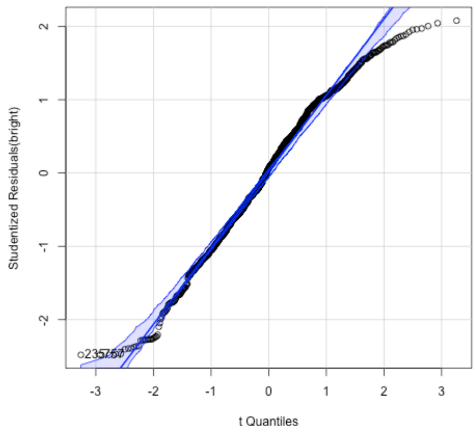

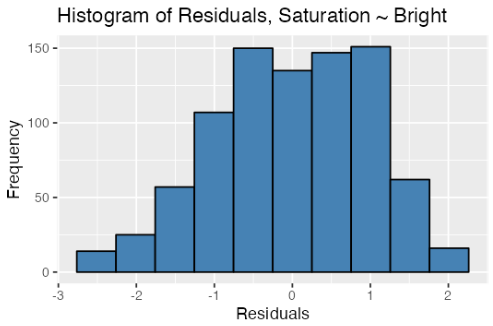

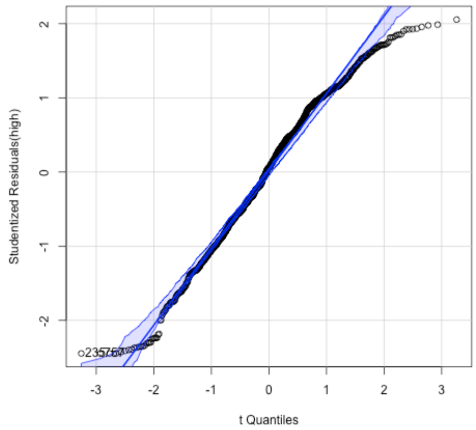

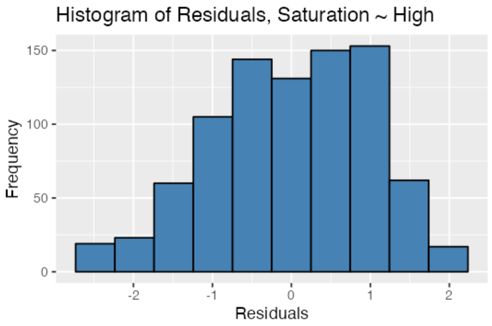

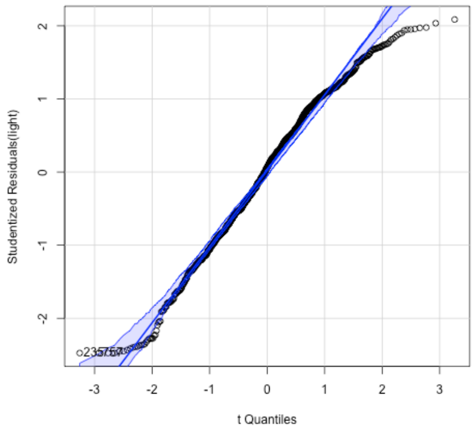

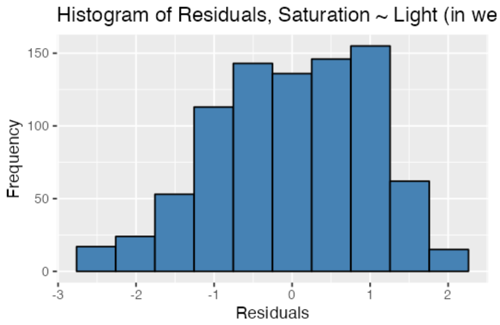

S2.2 . QQ Plots and Histograms: Experiment A Saturation *vs. X:*

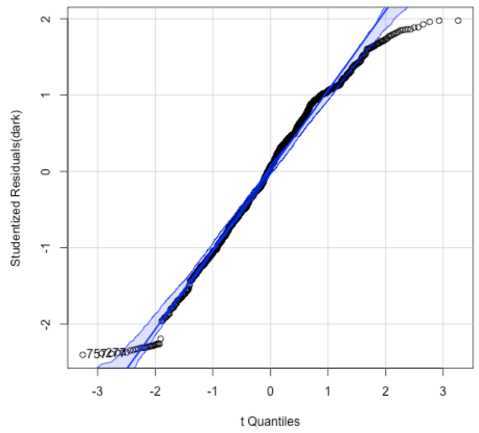

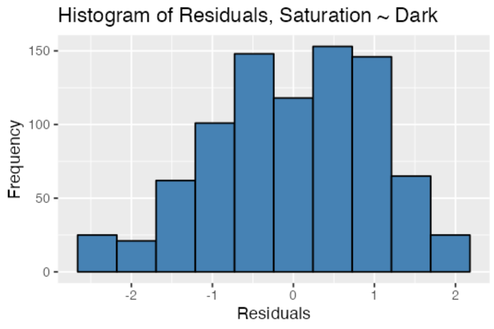

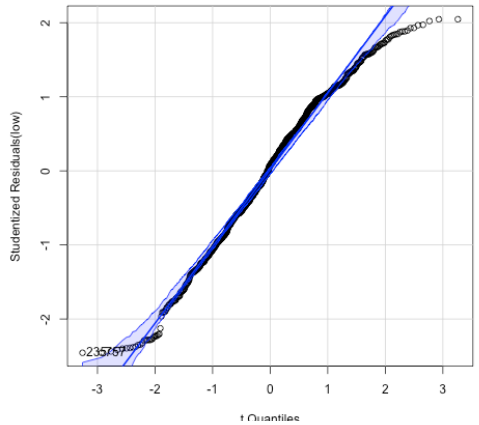

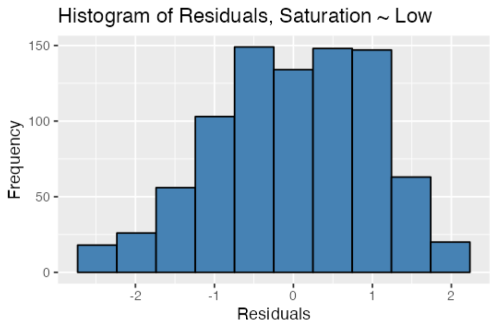

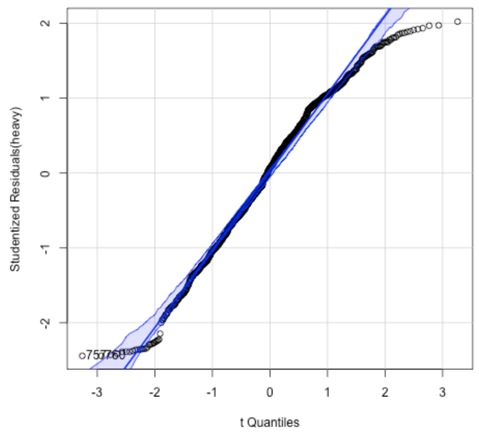

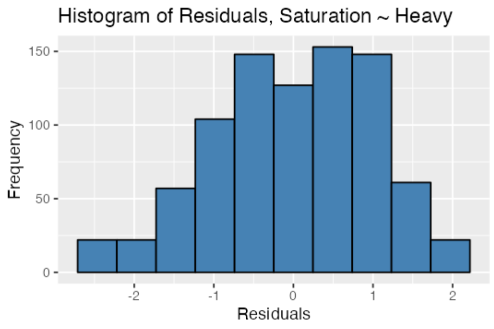

S2.2. QQ Plots and Histograms Experiment A cont'd: Saturation *vs. X:*

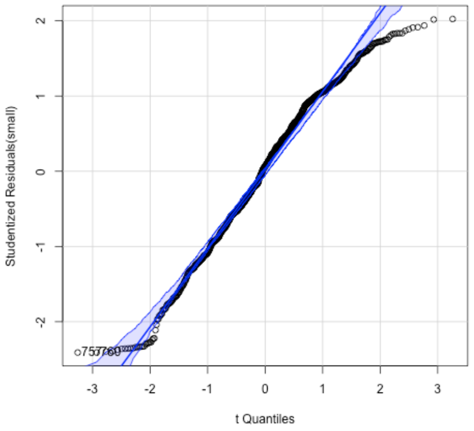

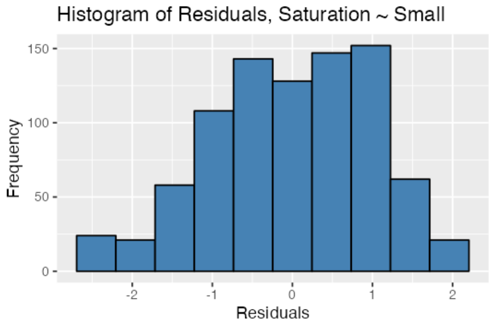

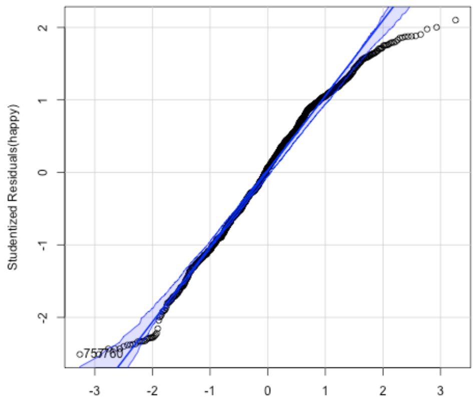

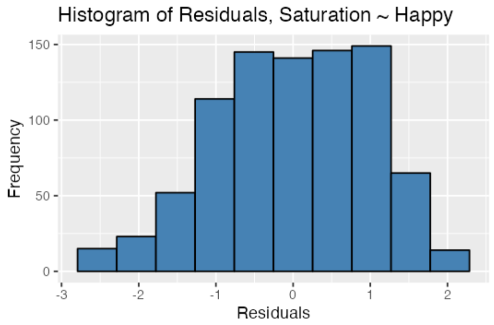

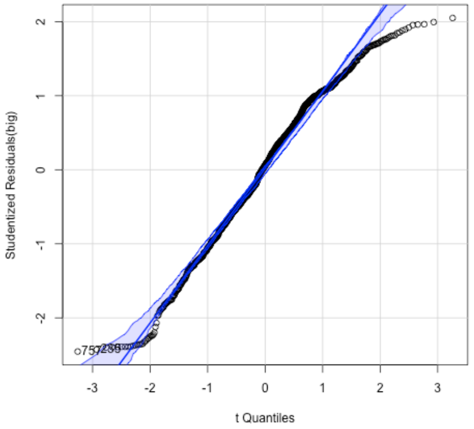

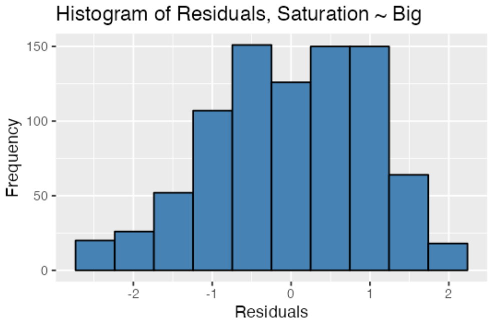

S2.2. QQ Plots and Histograms Experiment A cont'd: Saturation *vs. X:*

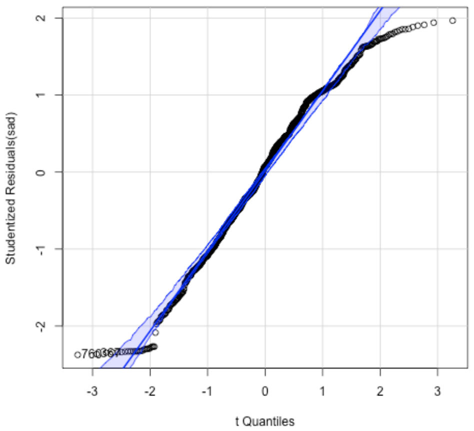

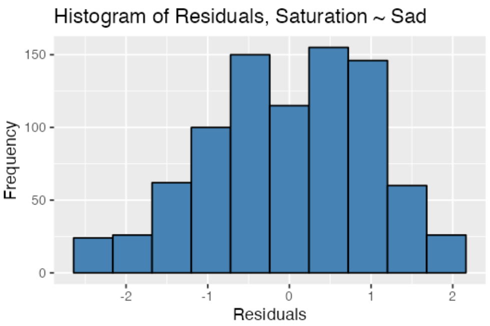

S2.3. QQ Plots and Histograms: Warm-Cool Index *vs. X:*

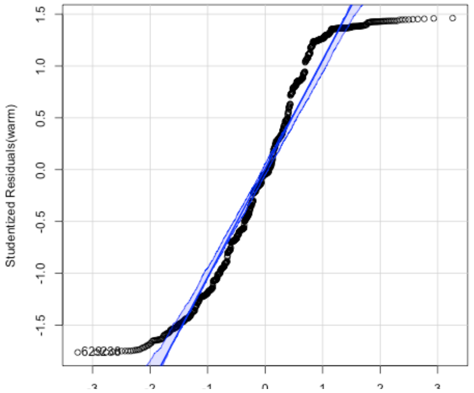

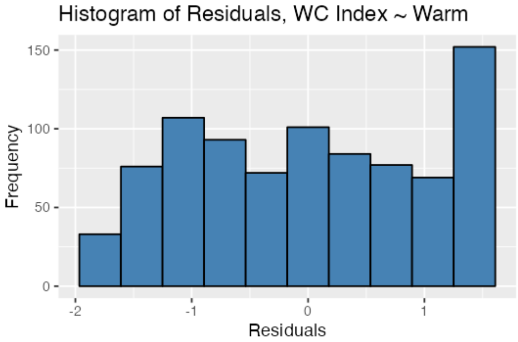

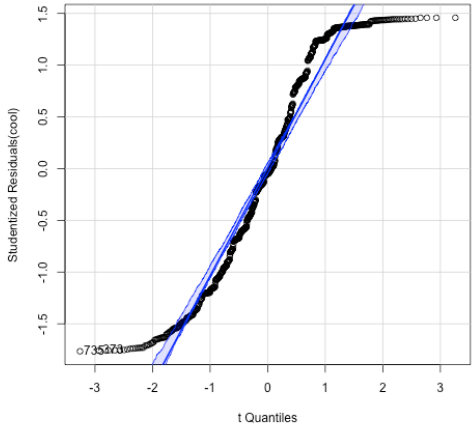

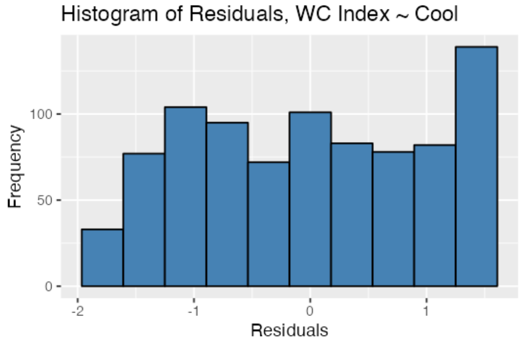

S3. Quantile-Quantile (QQ) Plots and Histograms of Residuals of Model Fits to Semantic Ratings in Experiment B (Orchestral Instruments).

S3.1. Lightness *vs.* *X*

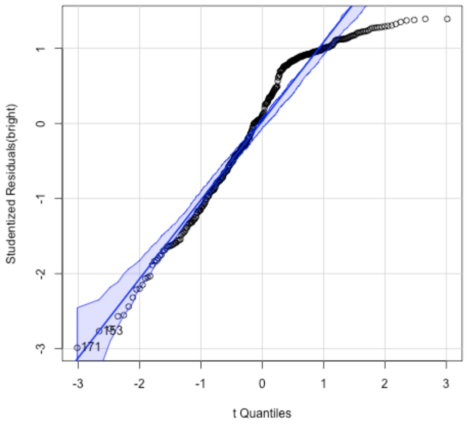

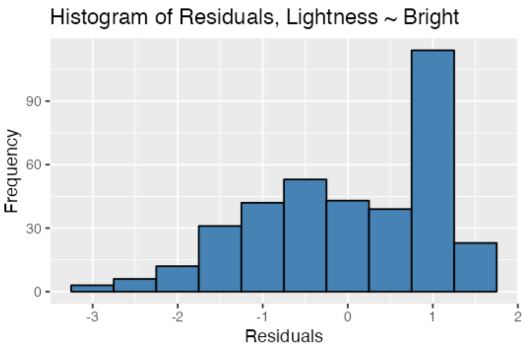

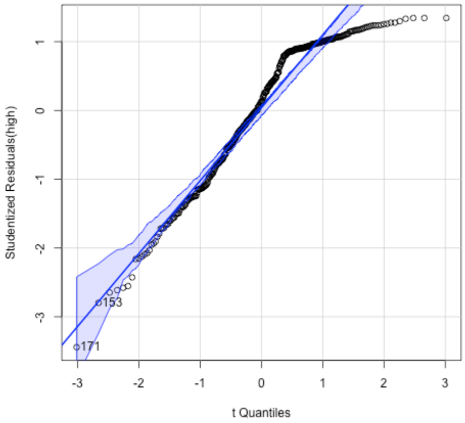

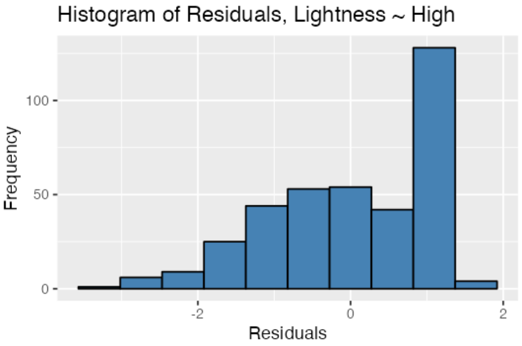

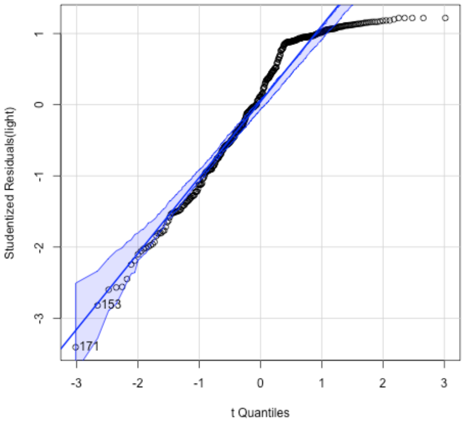

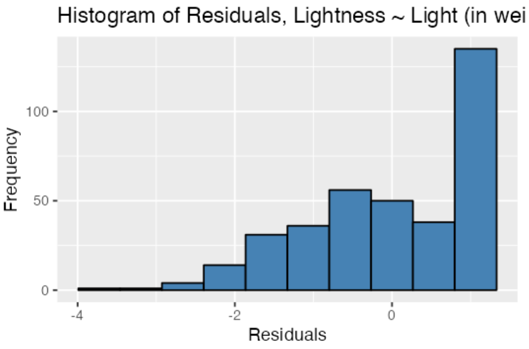

S3.1. QQ Plots and Histograms Experiment B cont'd: Lightness *vs. X:*

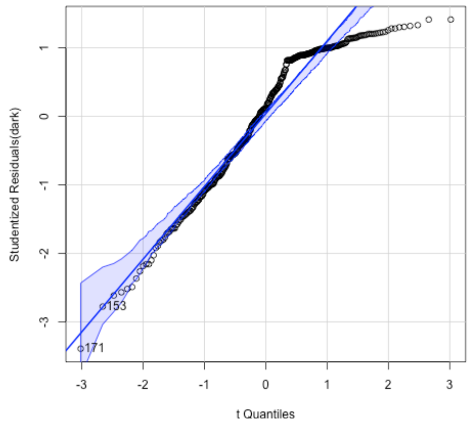

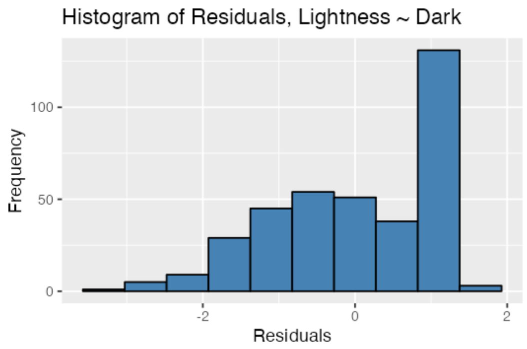

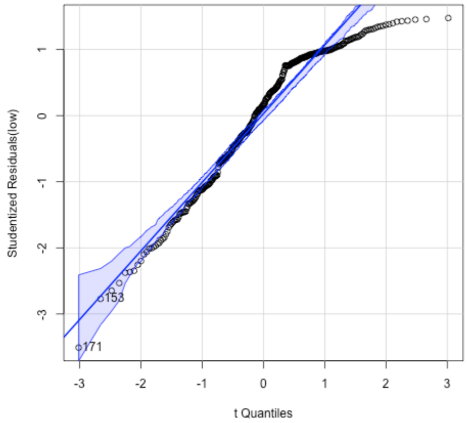

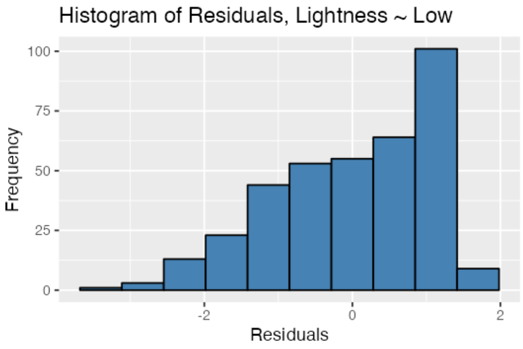

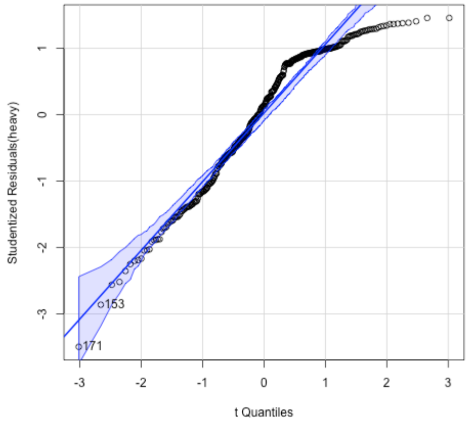

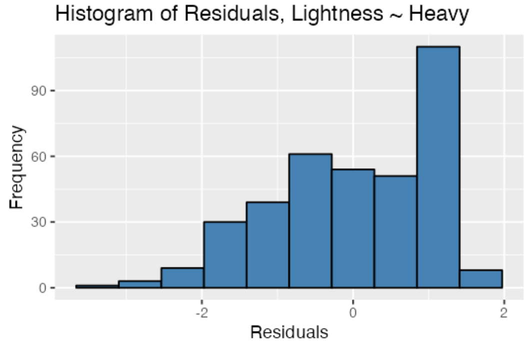

S3.1. QQ Plots and Histograms Experiment B cont'd: Lightness *vs. X:*

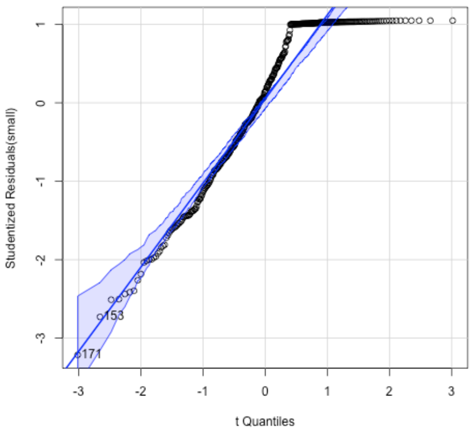

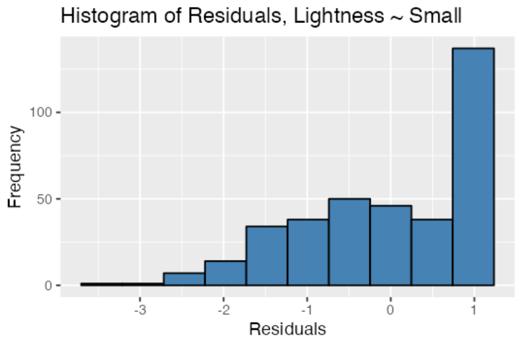

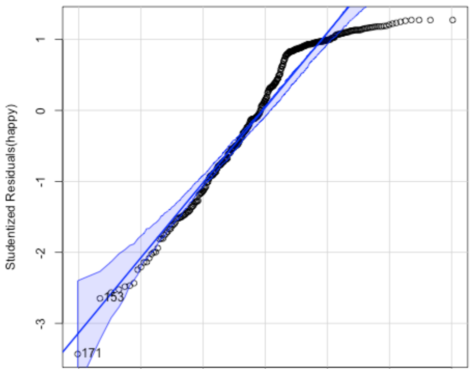

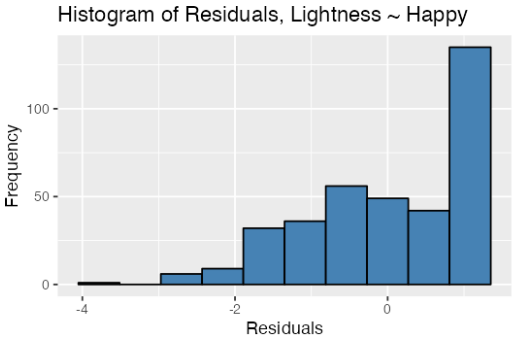

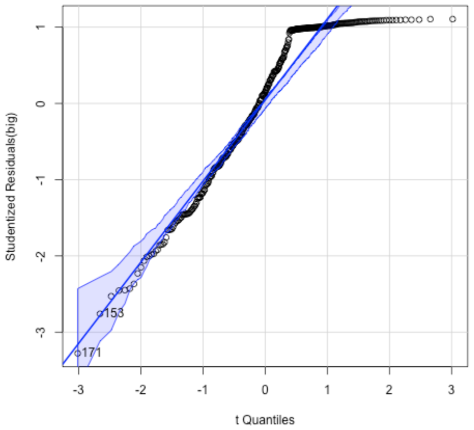

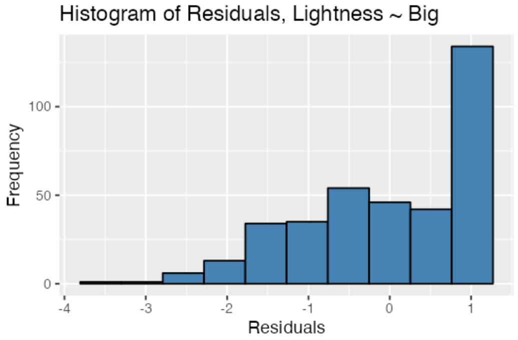

S3.1. QQ Plots and Histograms Experiment B cont'd: Lightness *vs. X:*

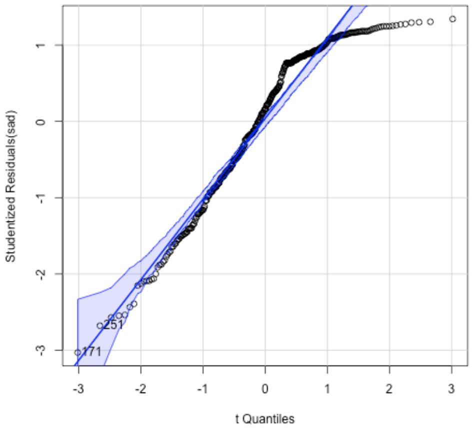

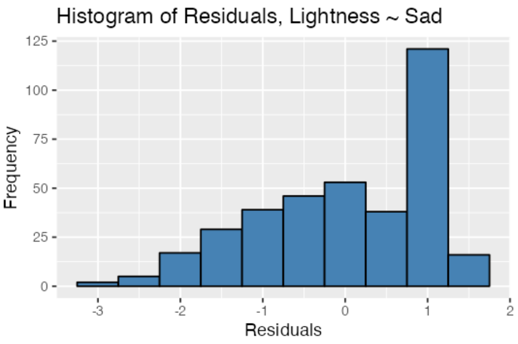

S3.2. QQ Plots and Histograms for Experiment B (Orchestral Instruments): Saturation *vs. X:*

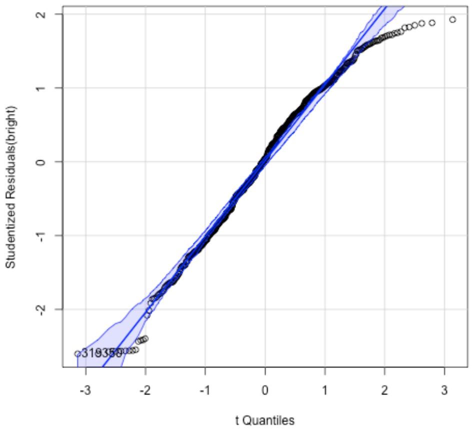

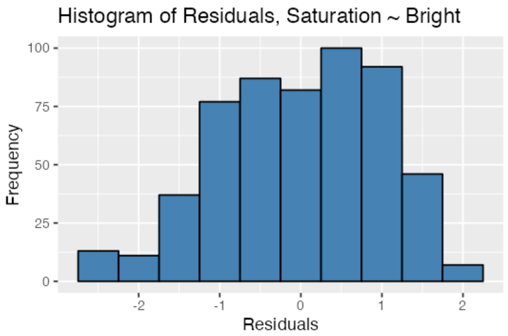

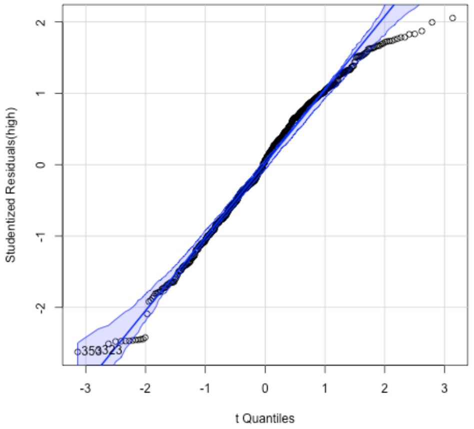

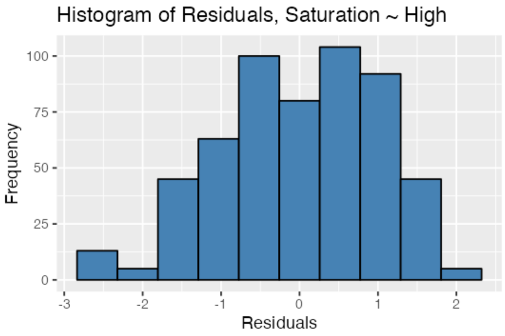

S3.2. QQ Plots and Histograms Experiment B cont'd: Saturation *vs. X:*

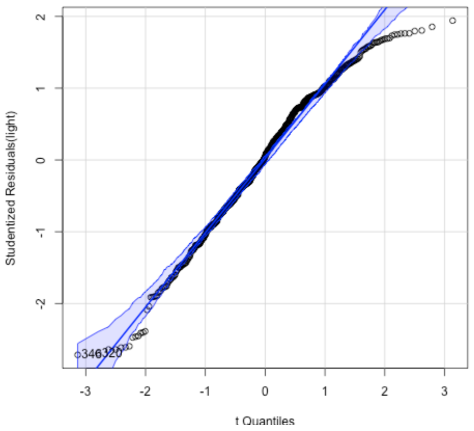

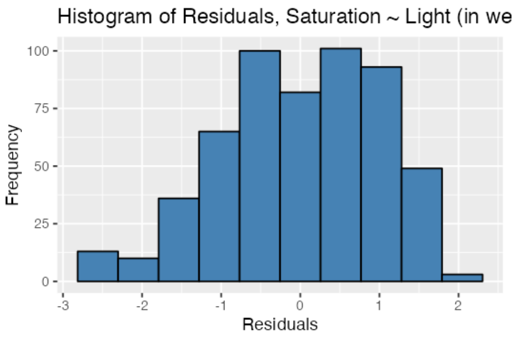

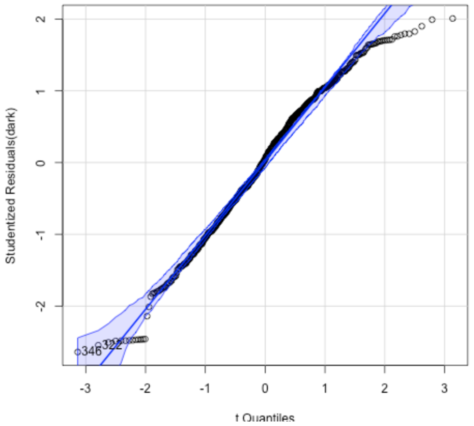

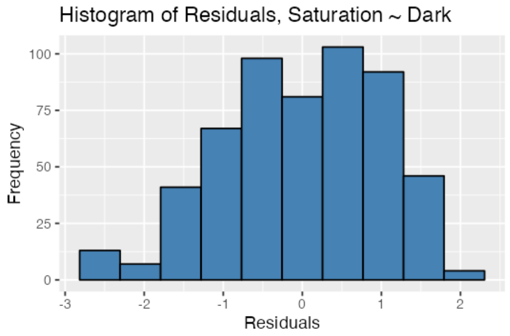

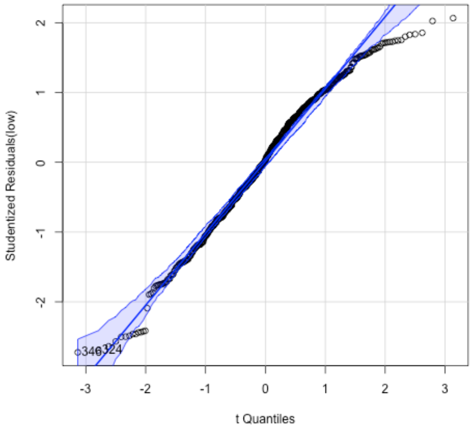

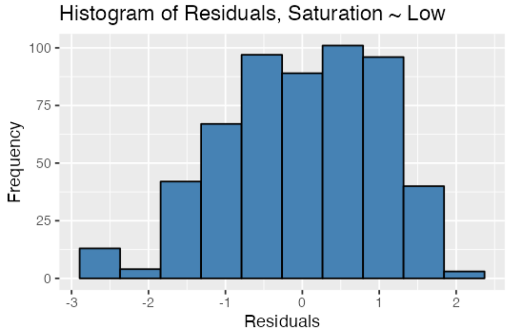

S3.2. QQ Plots and Histograms Experiment B cont’d: Saturation *vs. X:*

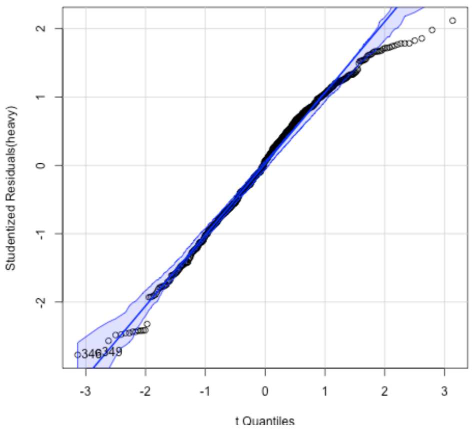

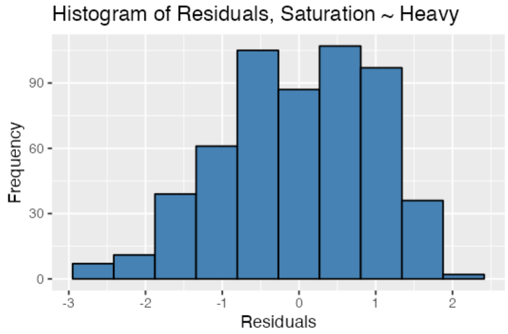

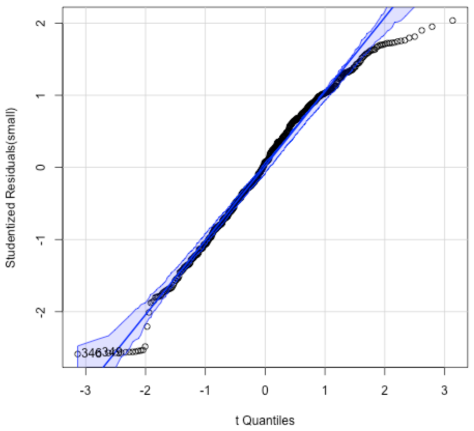

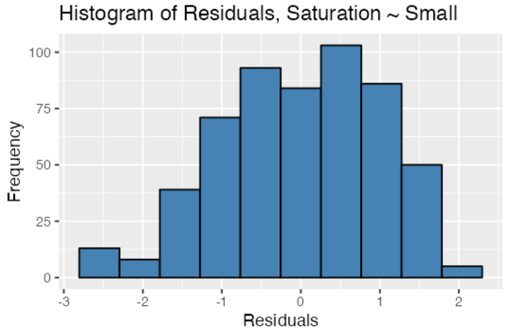

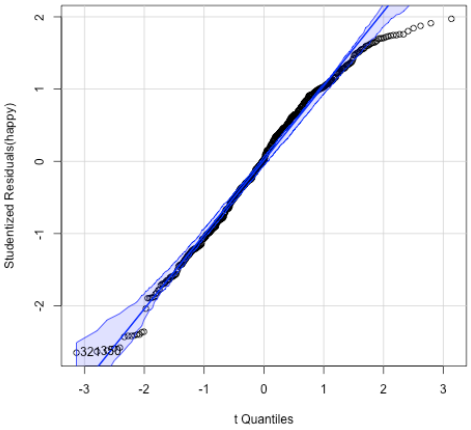

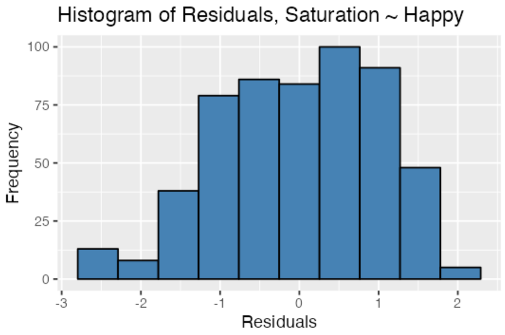

S3.2. QQ Plots and Histograms Experiment B cont'd: Saturation *vs. X:*

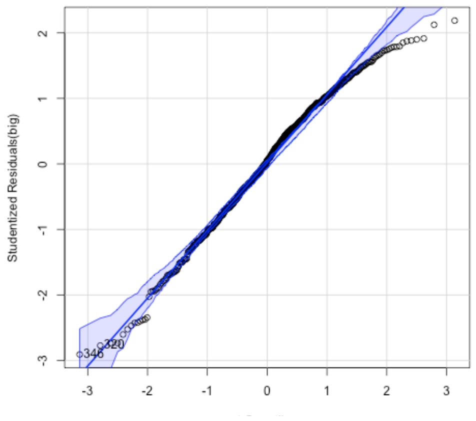

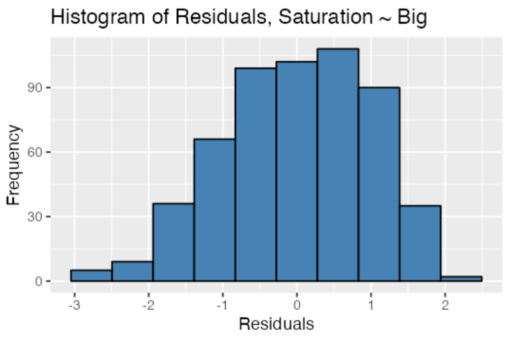

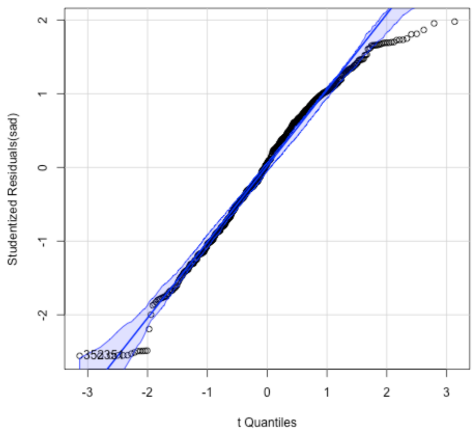

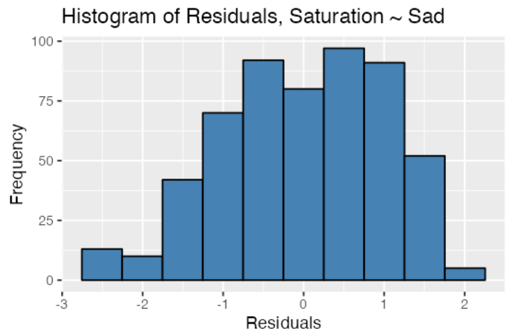

S3.3. QQ Plots and Histograms for Experiment B (Orchestral Instruments): Warm-Cool Index *vs. X:*

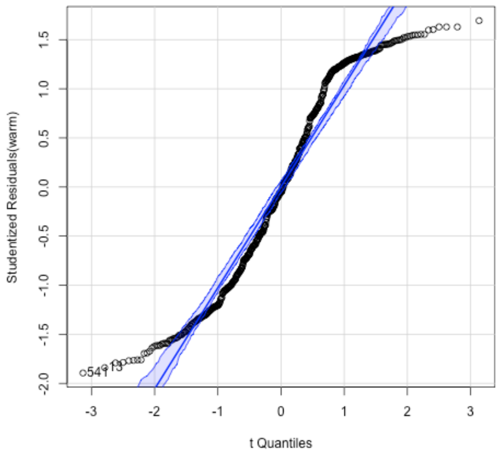

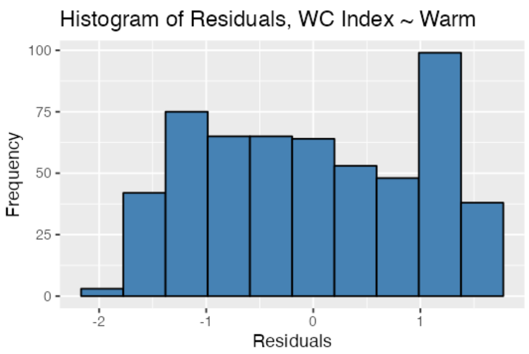

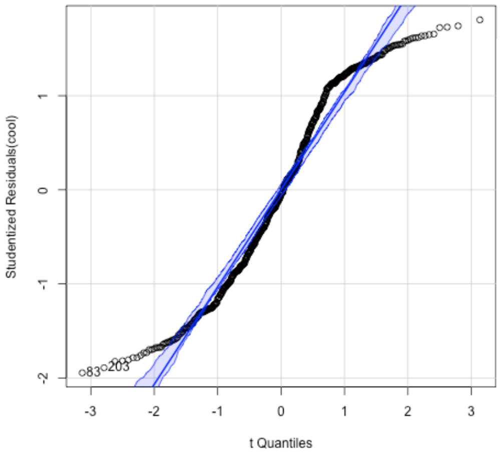

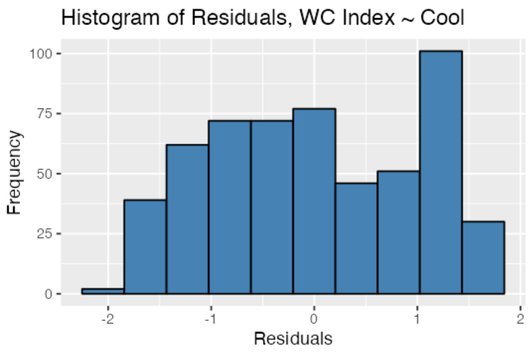

S4.1 Summary Statistics: Experiment A (Keyboards)

Lightness

	LMM			CLMM		
	p	marginal *R2*	conditional *R2*	*p*	marginal *R2*	conditional *R2*
**Bright**	<.001	.14	.21	<.001	.16	.23
**Dark**	<.001	.14	.19	<.001	.16	.21
**High**	<.001	.15	.19	<.001	.17	.21
**Low**	<.001	.16	.22	<.001	.18	.24
**Light in weight**	<.001	.15	.23	<.001	.18	.25
**Heavy**	<.001	.15	.21	<.001	.17	.23
**Small**	<.001	.10	.16	<.001	.12	.16
**Big**	<.001	.12	.17	<.001	.12	.17
**Happy**	<.001	.09	.14	<.001	.11	.14
**Sad**	<.001	.08	.12	<.001	.09	.12

Saturation

	LMM			CLMM		
	p	marginal *R2*	conditional *R2*	*p*	marginal *R2*	conditional *R2*
**Bright**	.01	.01	.14	.002	.01	.15
**Dark***	.11	.00	.14	.03	.01	.15
**High**	.01	.01	.14	.004	.01	.15
**Low**	.006	.01	.14	.005	.01	.15
**Light in weight**	.003	.01	.14	<.001	.02	.16
**Heavy**	.01	.01	.14	.003	.01	.15
**Small***	.05	.00	.14	.02	.01	.15
**Big**	.01	.01	.14	.003	.01	.15
**Happy**	.01	.01	.14	.002	.01	.15
**Sad**	.25	.00	.14	.19	.00	.14

Warm-Cool Index

	LMM			CLMM		
	p	marginal *R2*	conditional *R2*	*p*	marginal *R2*	conditional *R2*
**Warm**	.72	.00	.04	.45	.00	.04
**Cool**	.82	.00	.04	.81	.00	.04

* Note. *Dark* and *Small* in the saturation models for Experiment A are significant in CLMM only.

S4.2 Summary Statistics: Experiment B (Orchestral Instruments)

Lightness

	LMM			CLMM		
	p	marginal *R2*	conditional *R2*	*p*	marginal *R2*	conditional *R2*
**Bright**	.003	.02	.16	.005	.02	.18
**Dark**	.01	.02	.15	.007	.02	.17
**High**	.01	.02	.16	.005	.02	.18
**Low**	.001	.03	.16	<.001	.04	.18
**Light in weight**	.04	.01	.16	.02	.02	.18
**Heavy**	<.001	.03	.17	.004	.03	.18
**Small**	.62	.001	.15	.55	.00	.16
**Big**	.73	.000	.15	.71	.00	.16
**Happy**	.02	.01	.16	.007	.02	.18
**Sad**	.001	.03	.18	<.001	.03	.19

Saturation

	LMM			CLMM		
	p	marginal *R2*	conditional *R2*	*p*	marginal *R2*	conditional *R2*
**Bright**	.17	.00	.32	.16	.00	.26
**Dark**	.94	.00	.32	.99	.00	.26
**High**	.15	.00	.32	.24	.00	.27
**Low**	.21	.00	.32	.40	.00	.26
**Light in weight**	.02	.01	.33	.01	.01	.27
**Heavy**	.08	.01	.32	.06	.01	.26
**Small**	.12	.00	.33	.10	.01	.27
**Big**	<.001	.02	.23	<.001	.03	.29
**Happy**	.06	.01	.32	.07	.01	.26
**Sad**	.27	.00	.33	.38	.00	.26

Warm-Cool Index

	LMM			CLMM		
	p	marginal *R2*	conditional *R2*	*p*	marginal *R2*	conditional *R2*
**Warm**	.01	.01	.01	.02	.01	.01
**Cool**	<.001	.02	.02	<.001	.02	.02

S4.3 Experiment A (Keyboards) - Lightness, H1a

H1a. Semantic ratings on the terms bright, dark, high, low, light in weight, heavy, small, big, happy, and sad will predict the lightness of matched colors. Specifically, ratings on the terms bright, high, light in weight, small, and happy will be positively associated with lightness; ratings on dark, low, heavy, big, and sad will negatively correlate with lightness.

Note. Number of observations = 648, number of participants = 72. All variables have been scaled and centered.

Lightness ~ Bright + (1 | Participant)			
LMM			
**Fixed effects**	**Estimate**	**SE**	***p***
Intercept	-0.2	0.05	<.001
Bright	0.4	0.04	<.001
**Random effect**	**Variance**	**SD**	***p***
Participant	0.07	0.27	<.001
**Marginal R2**	.14		
**Conditional R2**	.21		
CLMM			
**Fixed effects**	**Estimate**	**SE**	***p***
Bright	0.84	0.09	<.001
**Random effect**	**Variance**	**SD**	***p***
Participant	0.29	0.54	.001
**Marginal R2**	.16		
**Conditional R2**	.23		

Lightness ~ Dark + (1 | Participant)			
LMM			
**Fixed effects**	**Estimate**	**SE**	***p***
Intercept	-0.18	0.05	<.001
Dark	-0.4	0.04	.01
**Random effect**	**Variance**	**SD**	***p***
Participant	0.05	0.23	.009
**Marginal R2**	.14		
**Conditional R2**	.19		
CLMM			
**Fixed effects**	**Estimate**	**SE**	***p***
Dark	-0.83	0.08	<.001
**Random effect**	**Variance**	**SD**	***p***
Participant	0.18	0.43	.03
**Marginal R2**	.16		
**Conditional R2**	.21		

S4.3 Experiment A (Keyboards) - Lightness, H1a, cont'd

Lightness ~ High + (1 | Participant)			
**LMM**			
**Fixed effects**	**Estimate**	**SE**	***p***
Intercept	-0.2	0.04	<.001
High	0.4	0.04	<.001
**Random effect**	**Variance**	**SD**	***p***
Participant	0.04	0.2	.04
**Marginal R2**	.15		
**Conditional R2**	.19		
**CLMM**			
**Fixed effects**	**Estimate**	**SE**	***p***
High	0.86	0.08	<.001
**Random effect**	**Variance**	**SD**	***p***
Participant	0.13	0.35	.10
**Marginal R2**	.17		
**Conditional R2**	.21		

Lightness ~ Low + (1 | Participant)			
**LMM**			
**Fixed effects**	**Estimate**	**SE**	***p***
Intercept	-0.19	0.05	<.001
Low	-0.41	0.04	<.001
**Random effect**	**Variance**	**SD**	***p***
Participant	0.07	0.26	.001
**Marginal R2**	.16		
**Conditional R2**	.22		
**CLMM**			
**Fixed effects**	**Estimate**	**SE**	***p***
Low	-0.88	0.09	<.001
**Random effect**	**Variance**	**SD**	***p***
Participant	0.24	0.49	.005
**Marginal R2**	.18		
**Conditional R2**	.24		

Lightness ~ Light in weight + (1 | Participant)			
**LMM**			
**Fixed effects**	**Estimate**	**SE**	***p***
Intercept	-0.19	0.05	<.001
Light in weight	0.42	0.04	<.001
**Random effect**	**Variance**	**SD**	***p***
Participant	0.08	0.28	<.001
**Marginal R2**	.15		
**Conditional R2**	.23		
**CLMM**			
**Fixed effects**	**Estimate**	**SE**	***p***
Light in weight	0.9	0.09	<.001
**Random effect**	**Variance**	**SD**	***p***
Participant	0.3	0.54	<.001
**Marginal R2**	.18		
**Conditional R2**	.25		

Lightness ~ Heavy + (1 | Participant)			
**LMM**			
**Fixed effects**	**Estimate**	**SE**	***p***
Intercept	-0.18	0.05	<.001
Heavy	-0.41	0.04	<.001
**Random effect**	**Variance**	**SD**	***p***
Participant	0.06	0.25	.002
**Marginal R2**	.15		
**Conditional R2**	.21		
**CLMM**			
**Fixed effects**	**Estimate**	**SE**	***p***
Heavy	-0.87	0.09	<.001
**Random effect**	**Variance**	**SD**	***p***
Participant	0.23	0.48	.009
**Marginal R2**	.17		
**Conditional R2**	.23		

S4.3 Experiment A (Keyboards) - Lightness, H1a, cont'd

Lightness ~ Small + (1 | Participant)			
LMM			
**Fixed effects**	**Estimate**	**SE**	***p***
Intercept	-0.19	0.05	<.001
Small	0.34	0.04	<.001
**Random effect**	**Variance**	**SD**	***p***
Participant	0.06	0.24	.009
**Marginal R2**	.10		
**Conditional R2**	.16		
CLMM			
**Fixed effects**	**Estimate**	**SE**	***p***
Small	0.69	0.09	<.001
**Random effect**	**Variance**	**SD**	***p***
Participant	0.18	0.42	.03
**Marginal R2**	.12		
**Conditional R2**	.16		

Lightness ~ Big + (1 | Participant)			
LMM			
**Fixed effects**	**Estimate**	**SE**	***p***
Intercept	-0.18	0.05	<.001
Big	-0.36	0.04	<.001
**Random effect**	**Variance**	**SD**	***p***
Participant	0.05	0.23	.02
**Marginal R2**	.12		
**Conditional R2**	.17		
CLMM			
**Fixed effects**	**Estimate**	**SE**	***p***
Big	-0.7	0.08	<.001
**Random effect**	**Variance**	**SD**	***p***
Participant	0.17	0.42	.03
**Marginal R2**	.12		
**Conditional R2**	.17		

Lightness ~ Happy + (1 | Participant)			
LMM			
**Fixed effects**	**Estimate**	**SE**	***p***
Intercept	-0.2	0.05	<.001
Happy	0.33	0.04	<.001
**Random effect**	**Variance**	**SD**	***p***
Participant	0.05	0.21	.03
**Marginal R2**	.09		
**Conditional R2**	.14		
CLMM			
**Fixed effects**	**Estimate**	**SE**	***p***
Happy	0.67	0.08	<.001
**Random effect**	**Variance**	**SD**	***p***
Participant	0.13	0.37	.08
**Marginal R2**	.11		
**Conditional R2**	.14		

Lightness ~ Sad + (1 | Participant)			
LMM			
**Fixed effects**	**Estimate**	**SE**	***p***
Intercept	-0.19	0.05	<.001
Sad	-0.31	0.04	<.001
**Random effect**	**Variance**	**SD**	***p***
Participant	0.04	0.21	.04
**Marginal R2**	.08		
**Conditional R2**	.12		
CLMM			
**Fixed effects**	**Estimate**	**SE**	***p***
Sad	-0.6	0.08	<.001
**Random effect**	**Variance**	**SD**	***p***
Participant	0.11	0.34	.13
**Marginal R2**	.09		
**Conditional R2**	.12		

S4.4 Experiment B (Orchestral Instruments) - Lightness, H1a

H1a. Semantic ratings on the terms bright, dark, high, low, light in weight, heavy, small, big, happy, and sad will predict the lightness of matched colors. Specifically, ratings on the terms bright, high, light in weight, small, and happy will be positively associated with lightness; ratings on dark, low, heavy, big, and sad will negatively correlate with lightness.

Note. Number of observations = 366, number of participants = 61. All variables have been scaled and centered.

Lightness ~ Bright + (1 | Participant)			
LMM			
**Fixed effects**	**Estimate**	**SE**	***p***
Intercept	-0.34	0.07	<.001
Bright	0.16	0.05	.003
**Random effect**	**Variance**	**SD**	***p***
Participant	0.15	0.39	<.001
**Marginal R2**	.02		
**Conditional R2**	.16		
CLMM			
**Fixed effects**	**Estimate**	**SE**	***p***
Bright	0.29	0.11	.005
**Random effect**	**Variance**	**SD**	***p***
Participant	0.63	0.79	<.001
**Marginal R2**	.02		
**Conditional R2**	.18		

Lightness ~ Dark + (1 | Participant)			
LMM			
**Fixed effects**	**Estimate**	**SE**	***p***
Intercept	-0.34	0.07	<.001
Dark	-0.13	0.05	.01
**Random effect**	**Variance**	**SD**	***p***
Participant	0.15	0.39	<.001
**Marginal R2**	.02		
**Conditional R2**	.15		
CLMM			
**Fixed effects**	**Estimate**	**SE**	***p***
Dark	-0.28	0.1	.007
**Random effect**	**Variance**	**SD**	***p***
Participant	0.6	0.77	<.001
**Marginal R2**	.02		
**Conditional R2**	.17		

S4.4 Experiment B (Orchestral Instruments) - Lightness, H1a, cont'd

Lightness ~ High + (1 | Participant)			
LMM			
**Fixed effects**	**Estimate**	**SE**	***p***
Intercept	-0.34	0.07	<.001
High	0.13	0.05	.01
**Random effect**	**Variance**	**SD**	***p***
Participant	0.16	0.4	<.001
**Marginal R2**	.02		
**Conditional R2**	.16		
CLMM			
**Fixed effects**	**Estimate**	**SE**	***p***
High	0.3	0.11	.005
**Random effect**	**Variance**	**SD**	***p***
Participant	0.63	0.79	<.001
**Marginal R2**	.02		
**Conditional R2**	.18		

Lightness ~ Low + (1 | Participant)			
LMM			
**Fixed effects**	**Estimate**	**SE**	***p***
Intercept	-0.35	0.07	<.001
Low	-0.18	0.06	.001
**Random effect**	**Variance**	**SD**	***p***
Participant	0.14	0.37	<.001
**Marginal R2**	.03		
**Conditional R2**	.16		
CLMM			
**Fixed effects**	**Estimate**	**SE**	***p***
Low	-0.38	0.11	<.001
**Random effect**	**Variance**	**SD**	***p***
Participant	0.57	0.75	<.001
**Marginal R2**	.04		
**Conditional R2**	.18		

Lightness ~ Light in weight + (1 | Participant)			
LMM			
**Fixed effects**	**Estimate**	**SE**	***p***
Intercept	-0.34	0.07	<.001
Light in weight	0.11	0.05	.04
**Random effect**	**Variance**	**SD**	***p***
Participant	0.16	0.41	<.001
**Marginal R2**	.01		
**Conditional R2**	.16		
CLMM			
**Fixed effects**	**Estimate**	**SE**	***p***
Light in weight	0.25	0.11	.02
**Random effect**	**Variance**	**SD**	***p***
Participant	0.65	0.81	<.001
**Marginal R2**	.02		
**Conditional R2**	.18		

Lightness ~ Heavy + (1 | Participant)			
LMM			
**Fixed effects**	**Estimate**	**SE**	***p***
Intercept	-0.34	0.07	<.001
Heavy	-0.18	0.05	<.001
**Random effect**	**Variance**	**SD**	***p***
Participant	0.15	0.39	<.001
**Marginal R2**	.03		
**Conditional R2**	.16		
CLMM			
**Fixed effects**	**Estimate**	**SE**	***p***
Heavy	-0.31	0.11	.004
**Random effect**	**Variance**	**SD**	***p***
Participant	0.61	0.78	<.001
**Marginal R2**	.03		
**Conditional R2**	.18		

S4.4 Experiment B (Orchestral Instruments) - Lightness, H1a, con't

Lightness ~ Small + (1 | Participant)			
**LMM**			
**Fixed effects**	**Estimate**	**SE**	***p***
Intercept	-0.34	0.07	<.001
Small	0.03	0.05	.62
**Random effect**	**Variance**	**SD**	***p***
Participant	0.17	0.41	<.001
**Marginal R2**	.00		
**Conditional R2**	.15		
**CLMM**			
**Fixed effects**	**Estimate**	**SE**	***p***
Small	0.06	0.1	.54
**Random effect**	**Variance**	**SD**	***p***
Participant	0.64	0.8	<.001
**Marginal R2**	.00		
**Conditional R2**	.16		

Lightness ~ Big + (1 | Participant)			
**LMM**			
**Fixed effects**	**Estimate**	**SE**	***p***
Intercept	-0.34	0.07	<.001
Big	-0.02	0.06	.73
**Random effect**	**Variance**	**SD**	***p***
Participant	0.16	0.4	<.001
**Marginal R2**	.00		
**Conditional R2**	.15		
**CLMM**			
**Fixed effects**	**Estimate**	**SE**	***p***
Big	-0.04	0.11	.72
**Random effect**	**Variance**	**SD**	***p***
Participant	0.62	0.79	<.001
**Marginal R2**	.00		
**Conditional R2**	.16		

Lightness ~ Happy + (1 | Participant)			
**LMM**			
**Fixed effects**	**Estimate**	**SE**	***p***
Intercept	-0.34	0.07	<.001
Happy	0.12	0.05	.02
**Random effect**	**Variance**	**SD**	***p***
Participant	0.16	0.4	<.001
**Marginal R2**	.01		
**Conditional R2**	.16		
**CLMM**			
**Fixed effects**	**Estimate**	**SE**	***p***
Happy	0.29	0.11	.007
**Random effect**	**Variance**	**SD**	***p***
Participant	0.64	0.8	<.001
**Marginal R2**	.02		
**Conditional R2**	.18		

Lightness ~ Sad + (1 | Participant)			
**LMM**			
**Fixed effects**	**Estimate**	**SE**	***p***
Intercept	-0.34	0.07	<.001
Sad	-0.17	0.05	.001
**Random effect**	**Variance**	**SD**	***p***
Participant	0.17	0.41	<.001
**Marginal R2**	.03		
**Conditional R2**	.18		
**CLMM**			
**Fixed effects**	**Estimate**	**SE**	***p***
Sad	-0.35	0.11	<.001
**Random effect**	**Variance**	**SD**	***p***
Participant	0.66	0.81	<.001
**Marginal R2**	.03		
**Conditional R2**	.19		

S4.5 Experiment A (Keyboards) - Saturation, H1b

H1b. Semantic ratings on the terms bright, dark, high, low, light in weight, heavy, small, big, happy, and sad are associated with saturation of matched colors. Specifically, ratings on the terms bright, high, light in weight, small, and happy will be negatively associated with saturation; ratings on dark, low, heavy, big, and sad will positively correlate with saturation.

Note. Number of observations = 864, number of participants = 96. All variables have been scaled and centered.

Saturation ~ Bright + (1 | Participant)			
LMM			
**Fixed effects**	**Estimate**	**SE**	***p***
Intercept	0.00	0.05	1
Bright	-0.08	0.03	.01
**Random effect**	**Variance**	**SD**	***p***
Participant	0.13	0.36	<.001
**Marginal R2**	0.01		
**Conditional R2**	0.14		
CLMM			
**Fixed effects**	**Estimate**	**SE**	***p***
Bright	-0.20	0.07	.002
**Random effect**	**Variance**	**SD**	***p***
Participant	0.53	0.73	<.001
**Marginal R2**	0.01		
**Conditional R2**	0.15		

Saturation ~ Dark + (1 | Participant)			
LMM			
**Fixed effects**	**Estimate**	**SE**	***p***
Intercept	0.00	0.05	1
Dark	0.05	0.03	.11
**Random effect**	**Variance**	**SD**	***p***
Participant	0.13	0.37	<.001
**Marginal R2**	0.00		
**Conditional R2**	0.14		
CLMM			
**Fixed effects**	**Estimate**	**SE**	***p***
Dark	0.14	0.07	.03
**Random effect**	**Variance**	**SD**	***p***
Participant	0.55	0.74	<.001
**Marginal R2**	0.01		
**Conditional R2**	0.15		

S4.5 Experiment A (Keyboards) - Saturation, H1b, cont'd

Saturation ~ High + (1 | Participant)			
LMM			
**Fixed effects**	**Estimate**	**SE**	***p***
Intercept	0.00	0.05	1
High	-0.08	0.03	.01
**Random effect**	**Variance**	**SD**	***p***
Participant	0.13	0.37	<.001
**Marginal R2**	0.01		
**Conditional R2**	0.14		
CLMM			
**Fixed effects**	**Estimate**	**SE**	***p***
High	-0.18	0.06	.004
**Random effect**	**Variance**	**SD**	***p***
Participant	0.55	0.74	<.001
**Marginal R2**	0.01		
**Conditional R2**	0.15		

Saturation ~ Low + (1 | Participant)			
LMM			
**Fixed effects**	**Estimate**	**SE**	***p***
Intercept	0.00	0.05	1
Low	0.09	0.03	.006
**Random effect**	**Variance**	**SD**	***p***
Participant	0.14	0.37	<.001
**Marginal R2**	0.01		
**Conditional R2**	0.14		
CLMM			
**Fixed effects**	**Estimate**	**SE**	***p***
Low	0.18	0.06	.005
**Random effect**	**Variance**	**SD**	***p***
Participant	0.55	0.74	<.001
**Marginal R2**	0.01		
**Conditional R2**	0.15		

Saturation ~ Light in weight + (1 | Participant)			
LMM			
**Fixed effects**	**Estimate**	**SE**	***p***
Intercept	0.00	0.05	1
Light in weight	-0.10	0.03	.003
**Random effect**	**Variance**	**SD**	***p***
Participant	0.13	0.37	<.001
**Marginal R2**	0.01		
**Conditional R2**	0.14		
CLMM			
**Fixed effects**	**Estimate**	**SE**	***p***
Light in weight	-0.24	0.07	<.001
**Random effect**	**Variance**	**SD**	***p***
Participant	0.56	0.75	<.001
**Marginal R2**	0.02		
**Conditional R2**	0.16		

Saturation ~ Heavy + (1 | Participant)			
LMM			
**Fixed effects**	**Estimate**	**SE**	***p***
Intercept	0.00	0.05	1
Heavy	0.08	0.03	.01
**Random effect**	**Variance**	**SD**	***p***
Participant	0.14	0.37	<.001
**Marginal R2**	0.01		
**Conditional R2**	0.14		
CLMM			
**Fixed effects**	**Estimate**	**SE**	***p***
Heavy	0.19	0.06	.003
**Random effect**	**Variance**	**SD**	***p***
Participant	0.55	0.74	<.001
**Marginal R2**	0.01		
**Conditional R2**	0.15		

S4.5 Experiment A (Keyboards) - Saturation, H1b, cont'd

Saturation ~ Small + (1 | Participant)			
LMM			
**Fixed effects**	**Estimate**	**SE**	***p***
Intercept	0.00	0.05	1
Small	-0.07	0.03	.05
**Random effect**	**Variance**	**SD**	***p***
Participant	0.13	0.37	<.001
**Marginal R2**	0.00		
**Conditional R2**	0.14		
CLMM			
**Fixed effects**	**Estimate**	**SE**	***p***
Small	-0.16	0.07	.02
**Random effect**	**Variance**	**SD**	***p***
Participant	0.54	0.74	<.001
**Marginal R2**	0.01		
**Conditional R2**	0.15		

Saturation ~ Big + (1 | Participant)			
LMM			
**Fixed effects**	**Estimate**	**SE**	***p***
Intercept	0.00	0.05	1
Big	0.08	0.03	.01
**Random effect**	**Variance**	**SD**	***p***
Participant	0.13	0.37	<.001
**Marginal R2**	0.01		
**Conditional R2**	0.14		
CLMM			
**Fixed effects**	**Estimate**	**SE**	***p***
Big	0.20	0.07	.003
**Random effect**	**Variance**	**SD**	***p***
Participant	0.55	0.74	<.001
**Marginal R2**	0.01		
**Conditional R2**	0.15		

Saturation ~ Happy + (1 | Participant)			
LMM			
**Fixed effects**	**Estimate**	**SE**	***p***
Intercept	0.00	0.05	1
Happy	-0.08	0.03	.01
**Random effect**	**Variance**	**SD**	***p***
Participant	0.13	0.36	<.001
**Marginal R2**	0.01		
**Conditional R2**	0.14		
CLMM			
**Fixed effects**	**Estimate**	**SE**	***p***
Happy	-0.20	0.07	.003
**Random effect**	**Variance**	**SD**	***p***
Participant	0.52	0.72	<.001
**Marginal R2**	0.01		
**Conditional R2**	0.15		

Saturation ~ Sad + (1 | Participant)			
LMM			
**Fixed effects**	**Estimate**	**SE**	***p***
Intercept	0.00	0.05	1
Sad	0.04	0.03	.25
**Random effect**	**Variance**	**SD**	***p***
Participant	0.13	0.37	<.001
**Marginal R2**	0.00		
**Conditional R2**	0.14		
CLMM			
**Fixed effects**	**Estimate**	**SE**	***p***
Sad	0.09	0.07	.20
**Random effect**	**Variance**	**SD**	***p***
Participant	0.53	0.73	<.001
**Marginal R2**	0.00		
**Conditional R2**	0.14		

S4.6 Experiment B (Orchestral Instruments) - Saturation, H1b

H1b. Semantic ratings on the terms bright, dark, high, low, light in weight, heavy, small, big, happy, and sad are associated with saturation of matched colors. Specifically, ratings on the terms bright, high, light in weight, small, and happy will be negatively associated with saturation; ratings on dark, low, heavy, big, and sad will positively correlate with saturation.

Note. Number of observations = 552, number of participants = 92. All variables have been scaled and centered.

Saturation ~ Bright + (1 | Participant)			
LMM			
**Fixed effects**	**Estimate**	**SE**	***p***
Intercept	0.00	0.07	1
Bright	0.05	0.04	.17
**Random effect**	**Variance**	**SD**	***p***
Participant	0.32	0.57	<.001
**Marginal R2**	0.00		
**Conditional R2**	0.32		
CLMM			
**Fixed effects**	**Estimate**	**SE**	***p***
Bright	0.12	0.09	.17
**Random effect**	**Variance**	**SD**	***p***
Participant	1.16	1.08	<.001
**Marginal R2**	0.00		
**Conditional R2**	0.27		

Saturation ~ Dark + (1 | Participant)			
LMM			
**Fixed effects**	**Estimate**	**SE**	***p***
Intercept	0.00	0.07	1
Dark	0.00	0.04	.95
**Random effect**	**Variance**	**SD**	***p***
Participant	0.32	0.57	<.001
**Marginal R2**	0.00		
**Conditional R2**	0.32		
CLMM			
**Fixed effects**	**Estimate**	**SE**	***p***
Dark	0.00	0.09	.99
**Random effect**	**Variance**	**SD**	***p***
Participant	1.17	1.08	<.001
**Marginal R2**	0.00		
**Conditional R2**	0.26		

S4.6 Experiment B (Orchestral Instruments) - Saturation, H1b, cont'd

Saturation ~ High + (1 | Participant)			
LMM			
**Fixed effects**	**Estimate**	**SE**	***p***
Intercept	0.00	0.07	1
High	-0.06	0.04	.15
**Random effect**	**Variance**	**SD**	***p***
Participant	0.32	0.57	<.001
**Marginal R2**	0.00		
**Conditional R2**	0.32		
CLMM			
**Fixed effects**	**Estimate**	**SE**	***p***
High	-0.10	0.09	.24
**Random effect**	**Variance**	**SD**	***p***
Participant	1.18	1.09	<.001
**Marginal R2**	0.00		
**Conditional R2**	0.27		

Saturation ~ Low + (1 | Participant)			
LMM			
**Fixed effects**	**Estimate**	**SE**	***p***
Intercept	0.00	0.07	1
Low	0.05	0.04	.21
**Random effect**	**Variance**	**SD**	***p***
Participant	0.32	0.57	<.001
**Marginal R2**	0.00		
**Conditional R2**	0.32		
CLMM			
**Fixed effects**	**Estimate**	**SE**	***p***
Low	0.07	0.09	.40
**Random effect**	**Variance**	**SD**	***p***
Participant	1.15	1.07	<.001
**Marginal R2**	0.00		
**Conditional R2**	0.26		

Saturation ~ Light in weight + (1 | Participant)			
LMM			
**Fixed effects**	**Estimate**	**SE**	***p***
Intercept	0.00	0.07	1
Light in weight	-0.09	0.04	.02
**Random effect**	**Variance**	**SD**	***p***
Participant	0.32	0.57	<.001
**Marginal R2**	0.00		
**Conditional R2**	0.33		
CLMM			
**Fixed effects**	**Estimate**	**SE**	***p***
Light in weight	-0.22	0.09	.01
**Random effect**	**Variance**	**SD**	***p***
Participant	1.17	1.08	<.001
**Marginal R2**	0.01		
**Conditional R2**	0.27		

Saturation ~ Heavy + (1 | Participant)			
LMM			
**Fixed effects**	**Estimate**	**SE**	***p***
Intercept	0.00	0.07	1
Heavy	0.07	0.04	.08
**Random effect**	**Variance**	**SD**	***p***
Participant	0.32	0.56	<.001
**Marginal R2**	0.01		
**Conditional R2**	0.32		
CLMM			
**Fixed effects**	**Estimate**	**SE**	***p***
Heavy	0.17	0.09	.06
**Random effect**	**Variance**	**SD**	***p***
Participant	1.15	1.07	<.001
**Marginal R2**	0.00		
**Conditional R2**	0.26		

S4.6 Experiment B (Orchestral Instruments) - Saturation, H1b, cont'd

Saturation ~ Small + (1 | Participant)			
LMM			
**Fixed effects**	**Estimate**	**SE**	***p***
Intercept	0.00	0.07	1
Small	-0.06	0.04	.12
**Random effect**	**Variance**	**SD**	***p***
Participant	0.33	0.57	<.001
**Marginal R2**	0.00		
**Conditional R2**	0.33		
CLMM			
**Fixed effects**	**Estimate**	**SE**	***p***
Small	-0.15	0.09	.10
**Random effect**	**Variance**	**SD**	***p***
Participant	1.18	1.08	<.001
**Marginal R2**	0.01		
**Conditional R2**	0.27		

Saturation ~ Big + (1 | Participant)			
LMM			
**Fixed effects**	**Estimate**	**SE**	***p***
Intercept	0.00	0.07	1
Big	0.01	0.04	<.001
**Random effect**	**Variance**	**SD**	***p***
Participant	0.31	0.56	<.001
**Marginal R2**	0.02		
**Conditional R2**	0.34		
CLMM			
**Fixed effects**	**Estimate**	**SE**	***p***
Big	0.36	0.09	<.001
**Random effect**	**Variance**	**SD**	***p***
Participant	1.18	1.09	<.001
**Marginal R2**	0.03		
**Conditional R2**	0.29		

Saturation ~ Happy + (1 | Participant)			
LMM			
**Fixed effects**	**Estimate**	**SE**	***p***
Intercept	0.00	0.07	1
Happy	0.08	0.04	.06
**Random effect**	**Variance**	**SD**	***p***
Participant	0.32	0.57	<.001
**Marginal R2**	0.00		
**Conditional R2**	0.32		
CLMM			
**Fixed effects**	**Estimate**	**SE**	***p***
Happy	0.16	0.09	.07
**Random effect**	**Variance**	**SD**	***p***
Participant	1.16	1.08	<.001
**Marginal R2**	0.01		
**Conditional R2**	0.27		

Saturation ~ Sad + (1 | Participant)			
LMM			
**Fixed effects**	**Estimate**	**SE**	***p***
Intercept	0.00	0.07	1
Sad	-0.04	0.04	.27
**Random effect**	**Variance**	**SD**	***p***
Participant	0.33	0.57	<.001
**Marginal R2**	0.00		
**Conditional R2**	0.32		
CLMM			
**Fixed effects**	**Estimate**	**SE**	***p***
Sad	-0.08	0.09	.38
**Random effect**	**Variance**	**SD**	***p***
Participant	1.17	1.08	<.001
**Marginal R2**	0.00		
**Conditional R2**	0.26		

S4.7 Warm/Cool, H1c

Experiment A (Keyboards) - Warm/Cool

H1c. Semantic ratings on the terms warm and cool are associated with perceived warmth/coolness of matched colors.

Note. Number of observations = 864, number of participants = 96. All variables have been scaled and centered.

Warm-Cool Index ~ Warm + (1 | Participant)			
LMM			
**Fixed effects**	**Estimate**	**SE**	***p***
Intercept	0.00	0.04	1
Warm	0.01	0.03	.72
**Random effect**	**Variance**	**SD**	***p***
Participant	0.04	0.19	.05
**Marginal R2**	0.00		
**Conditional R2**	0.04		
CLMM			
**Fixed effects**	**Estimate**	**SE**	***p***
Warm	0.05	0.06	.45
**Random effect**	**Variance**	**SD**	***p***
Participant	0.15	0.39	.02
**Marginal R2**	0.00		
**Conditional R2**	0.04		

Experiment B (Orchestral Instruments) - Warm/Cool

Note. Number of observations = 552, number of participants = 92. All variables have been scaled and centered.

Warm-Cool Index ~ Warm + (1 | Participant)			
LMM			
**Fixed effects**	**Estimate**	**SE**	***p***
Intercept	0.00	0.04	1
Warm	0.10	0.04	.01
**Random effect**	**Variance**	**SD**	***p***
Participant	0.00	0.00	1
**Marginal R2**	0.01		
**Conditional R2**	0.01		
CLMM			
**Fixed effects**	**Estimate**	**SE**	***p***
Warm	0.18	0.08	.02
**Random effect**	**Variance**	**SD**	***p***
Participant	0.00	0.00	1
**Marginal R2**	0.01		
**Conditional R2**	0.01		

S4.7 Warm/Cool, H1c, cont'd

Warm-Cool Index ~ Cool + (1 | Participant)			
LMM			
**Fixed effects**	**Estimate**	**SE**	***p***
Intercept	0.00	0.04	1
Cool	0.01	0.03	.82
**Random effect**	**Variance**	**SD**	***p***
Participant	0.04	0.19	.05
**Marginal R2**	0.00		
**Conditional R2**	0.04		
CLMM			
**Fixed effects**	**Estimate**	**SE**	***p***
Cool	0.01	0.06	.81
**Random effect**	**Variance**	**SD**	***p***
Participant	0.15	0.38	.02
**Marginal R2**	0.00		
**Conditional R2**	0.04		

Warm-Cool Index ~ Cool + (1 | Participant)			
LMM			
**Fixed effects**	**Estimate**	**SE**	***p***
Intercept	0.00	0.04	1
Cool	-0.20	0.04	<.001
**Random effect**	**Variance**	**SD**	***p***
Participant	0.00	0.00	1
**Marginal R2**	0.02		
**Conditional R2**	0.02		
CLMM			
**Fixed effects**	**Estimate**	**SE**	***p***
Cool	-0.26	0.08	<.001
**Random effect**	**Variance**	**SD**	***p***
NA	NA	NA	
**Marginal R2**	0.02		
**Conditional R2**	0.02		

Note. Variance-covariance matrix was not definable with random effect for the CLMM; model is reported without random effect. However, R2 is calculated from the model with random effect included.

S4.8 Color Matching, H2 & H3

H2. When asked to match timbres with colors, lightness, saturation, and warmth/coolness of participants' color choices will vary systematically across different musical instrument timbres.

H3. When asked to match timbres with colors, lightness, saturation, and warmth/coolness of participants' color choices will vary systematically with pitch register. Higher pitch register will result in increased lightness and decreased saturation.

Experiment A (Keyboards) - Instrument and Pitch Register

Lightness ~ Pitch Register * Keyboard Type + (1 | Participant)

Type III Analysis of Variance Table with Satterthwaite's method

	Sum Sq	Mean Sq	Num DF	Den DF	*F*	*p*
**Pitch Register**	184.90	92.45	2	568	131.18	<.001
**Keyboard Type**	7.59	3.80	2	568	5.39	.004
**Pitch Register * Keyboard Type**	0.70	0.17	4	568	0.25	.91
**Marginal R2**	0.28					
**Conditional R2**	0.34					

Saturation ~ Pitch Register * Keyboard Type + (1 | Participant)

Type III Analysis of Variance Table with Satterthwaite's method

	Sum Sq	Mean Sq	Num DF	Den DF	*F*	*p*
**Pitch Register**	20.59	10.30	2	760	12.43	<.001
**Keyboard Type**	13.31	6.65	2	760	8.03	<.001
**Pitch Register * Keyboard Type**	3.73	0.93	4	760	1.13	.34
**Marginal R2**	0.04					
**Conditional R2**	0.18					

Warm-Cool Index ~ Pitch Register * Keyboard Type + (1 | Participant)

Type III Analysis of Variance Table with Satterthwaite's method

	Sum Sq	Mean Sq	Num DF	Den DF	*F*	*p*
**Pitch Register**	21.01	10.50	2	760	11.74	<.001
**Keyboard Type**	35.27	17.64	2	760	19.71	<.001
**Pitch Register * Keyboard Type**	4.74	1.19	4	760	1.33	.26
**Marginal R2**	0.07					
**Conditional R2**	0.11					

S4.8 Color Matching, H2 & H3, cont'd

Experiment B (Orchestral Instruments) - Instrument

Lightness ~ Instrument Type + (1 | Participant)						
Type III Analysis of Variance Table with Satterthwaite's method						
	**Sum Sq**	**Mean Sq**	**Num DF**	**Den DF**	***F***	***p***
**Instrument Type**	9.30	1.86	5	300	1.98	.08
**Marginal R2**	0.02					
**Conditional R2**	0.17					

Saturation ~ Instrument Type + (1 | Participant)						
Type III Analysis of Variance Table with Satterthwaite's method						
	**Sum Sq**	**Mean Sq**	**Num DF**	**Den DF**	***F***	***p***
**Instrument Type**	9.65	1.93	5	455	2.89	.01
**Marginal R2**	0.02					
**Conditional R2**	0.34					

Warm-Cool Index ~ Instrument Type + (1 | Participant)						
Type III Analysis of Variance Table with Satterthwaite's method						
	**Sum Sq**	**Mean Sq**	**Num DF**	**Den DF**	***F***	***p***
**Instrument Type**	14.80	2.96	5	455	3.03	.01
**Marginal R2**	0.03					
**Conditional R2**	0.03					
